# Engaging stakeholders in the co-development of programs or interventions using Intervention Mapping: A scoping review

**DOI:** 10.1371/journal.pone.0209826

**Published:** 2018-12-26

**Authors:** Umair Majid, Claire Kim, Albina Cako, Anna R. Gagliardi

**Affiliations:** Toronto General Hospital Research Institute, University Health Network, Toronto, Ontario, Canada; University of New South Wales, AUSTRALIA

## Abstract

**Background:**

Health care innovations tailored to stakeholder context are more readily adopted. This study aimed to describe how Intervention Mapping (IM) was used to design health care innovations and how stakeholders were involved.

**Methods:**

A scoping review was conducted. MEDLINE, EMBASE, Cochrane Library, Scopus and Science Citation Index were searched from 2008 to November 2017. English language studies that used or cited Intervention Mapping were eligible. Screening and data extraction were done in triplicate. Summary statistics were used to describe study characteristics, IM steps employed, and stakeholder involvement.

**Results:**

A total of 852 studies were identified, 449 were unique, and 333 were excluded based on title and abstracts, 116 full-text articles were considered and 61 articles representing 60 studies from 13 countries for a variety of clinical issues were included. The number of studies published per year increased since 2008 and doubled in 2016 and 2017. The majority of studies employed multiple research methods (76.7%) and all 6 IM steps (73.3%). Resulting programs/interventions were single (55.4%) or multifaceted (46.4%), and 60.7% were pilot-tested. Programs or interventions were largely educational material or meetings, and were targeted to patients (70.2%), clinicians (14.0%) or both (15.8%). Studies provided few details about current or planned evaluation. Of the 4 (9.3%) studies that reported impact or outcomes, 3 achieved positive improvements in patient or professional behaviour or patient outcomes. Many studies (28.3%) did not involve stakeholders. Those that did (71.7%) often involved a combination of patients, clinicians, and community organizations. However, less than half (48.8%) described how they were engaged. Most often stakeholders were committee members and provide feedback on program or intervention content or format.

**Conclusions:**

It is unclear if use of IM or stakeholder engagement in IM consistently results in effective programs or interventions. Those employing IM should report how stakeholders were involved in each IM step and how involvement influenced program or intervention design. They should also report the details or absence of planned evaluation. Future research should investigate how to optimize stakeholder engagement in IM, and whether use of IM itself or stakeholder engagement in IM are positively associated with effective programs or interventions.

## Background

Health care innovations such as guidelines, procedures, treatments, technology or programs have limited impact on health service delivery and health outcomes unless they are actively implemented. A crucial step in implementation planning is identifying determinants of innovation use including barriers or facilitators [1]. Flottorp consolidated several frameworks to generate a checklist of 57 potential determinants of the use of innovations that were organized in seven domains: guideline factors, individual health professional factors, patient factors, professional interactions, incentives and resources, capacity for organizational change, and social, political, and legal factors [2]. Knowledge of determinants can be collected through interviews, focus groups, surveys or observation of behaviour among target users of the innovation [3]. This information can then be used to select and tailor interventions that are most likely to promote and support use of the innovation. Research shows that interventions selected and tailored to address identified determinants were more likely to improve professional practice compared with either no intervention or simple dissemination of information about the innovation [4]. Suitable interventions can be identified by mapping pre-identified determinants in existing taxonomies of behaviour change interventions such as the Expert Recommendations for Implementing Change compendium [5], or by using resources such as the Theoretical Domains Framework [6].

Despite the guidance provided by these useful processes and tools, there is no consistently reliable mechanism for matching determinants to interventions that guarantees the successful implementation and use of innovations [7,8]. One approach for optimizing the selection, tailoring, implementation and impact of interventions is to engage target users of the innovation in implementation planning. They can provide important insight on how the determinants influence practice, which interventions are the best fit, and the most practical way to implement them. This approach, sometimes referred to as participatory research, engaged scholarship or mode 2 research, and more recently as integrated knowledge translation (IKT), recognizes that intervention developer-user collaboration can enhance the relevance and use of innovations [9]. However, such collaborations are challenging to develop and maintain. Our scoping review of 13 IKT-based studies published between 2005 and 2014 identified numerous barriers including differing priorities among participants, lack of skill or experience with IKT, unclear roles and goals, lack of incentives to participate, little continuity due to infrequent participation or turnover, and lack of resources [9]. Even when well-funded, the impact of IKT collaborations on the use of innovations, and improvements in health service delivery and health outcomes has been modest. For example, large-scale investment to foster IKT through the Collaborations for Leadership in Applied Health Research and Care (CLAHRC) in England [10] and Academic Collaborative Centres (ACC) for Public Health in the Netherlands [11] did not appear to eliminate barriers or lead to greater output or use of innovations. Hence, further knowledge is needed on how to achieve and support IKT. In particular, ongoing research should identify processes that are conducive to interaction among the developers and users of innovations, and appropriate roles for innovation users in the implementation planning process.

Intervention Mapping (IM) is a process that is specifically meant to engage stakeholders in developing interventions that address pre-identified determinants through six steps: assess needs and barriers, establish objectives, select theory-informed interventions, design and pilot-test the intervention, implement and assess fidelity of the intervention, and evaluate the impact of the intervention [12,13]. First published in 1998, IM is not a new process. However it is unclear, given growing recognition of the need to engage stakeholders in implementation planning, if and how IM has been used to co-develop innovations. The purpose of this study was to review the health care literature to describe how stakeholders were involved in co-developing programs or interventions including their roles and processes employed to engage them. The findings could generate insight on how to optimize IM and IKT in IM.

## Methods

### Approach

Given the aim, which was to describe the characteristics of published research that employed IM, a scoping review was conducted, comprised of five steps: scoping, searching, screening, data extraction and data analysis [14]. Similar in rigour to a systematic review, the purpose of a scoping review is to examine the nature of research activity for a particular topic, the volume of which cannot be known without such an assessment. Such reviews do not synthesize outcomes reported across studies or assess their methodological quality, as is customary of systematic reviews, nor do they assume a theoretical stance. However, they can assess what is known about a specific topic, identify if sufficient research is available to conduct a future systematic review, and/or reveal knowledge gaps that warrant future research. The Preferred Reporting Items for Systematic Reviews and Meta-Analyses (PRISMA) criteria guided reporting of the methods and findings (S1 Table) [15]. Data were publicly available so institutional review board approval was not necessary. A protocol for this review was not registered.

### Scoping

Relevant literature was first explored to become familiar with the IM literature and refine study methods. To do this, MEDLINE was searched using only the term “intervention mapping” as a keyword. AC and ARG independently screened titles and abstracts and discussed the findings. This knowledge was used to plan a more comprehensive search strategy and to generate eligibility criteria based on the PICO (participants, intervention, comparisons, outcomes) framework, as follows.

*Populations* referred to health care researchers, policy-makers or managers, clinicians, allied health staff, patients, care partners or consumers involved in the IM process that was used to plan an intervention relevant to screening, prevention, treatment (including drug/non-drug treatment, counseling, education, rehabilitation), follow-up, supportive care or palliative care offered in any health care setting. The *intervention* of interest was the IM process or any of its six steps (S2 Table). Relevant publications may have reported the entirety of the six-step IM process or several intervention planning steps (study type #1); often implementation and evaluation steps (study type #2) were reported in separate publications. *Comparisons* were based on study type. Type #1 studies tended to be qualitative studies, qualitative single or multiple case studies, or mixed methods studies that described the methods and outcomes of one or more IM steps. Type #2 studies tended to compare single or multiple interventions developed through the IM process either alone or compared with no intervention (sometimes described as “usual care” or “control”), or in comparison with another type of single or multiple intervention. The research design of type #2 studies included randomized or pragmatic controlled trials, observational studies (retrospective, prospective or before-after cohort studies), surveys, qualitative research (interviews, focus groups) or mixed methods research. *Outcomes* included but were not limited to IM steps used, type of innovation users involved and how, and with what outcome, meaning were interventions or programs developed, were they implemented, and did they influence health care delivery or patient health.

Studies were not eligible if they examined the effectiveness of clinical interventions (tests, procedures, treatment); used IM as a framework to extract data from research studies or documents such as practice guidelines but not to develop an intervention; used IM to develop policy or system-level interventions; used IM to develop health-related interventions applied in sport or school settings; or were publications in the form of editorials, letters, commentaries, protocols, meeting abstracts or proceedings, or conceptual analyses of IM. Systematic reviews were not eligible but were used to identify eligible primary studies.

### Searching

A comprehensive literature search was conducted. MEDLINE, EMBASE, Cochrane Library and Scopus were searched on April 21, 2016 for studies published since 1998, the year in which the IM process was first published [12], for the phrase “intervention mapping” as a keyword. Science Citation Index was searched for all subsequently published studies that referenced the 1998 IM publication [12]. The search was subsequently updated. MEDLINE, EMBASE and Science Citation Index were searched on November 8, 2017 for studies published in 2016 and 2017. The references of all eligible studies were scanned to identify additional eligible articles.

### Screening

AC and ARG independently screened all titles and abstracts of the initial search according to specified eligibility criteria. For the updated search, CK and ARG independently screened the title and abstract of the first 25 search results, then compared and discussed discrepancies, and how to interpret and apply the eligibility criteria. CK and ARG screened another 25 titles and abstracts and achieved congruent decisions regarding eligibility; henceforth, CK screened remaining titles and abstracts. At this stage we retained all studies published from 2008 and later so that findings and corresponding implications would be relevant to more recent IKT conduct and reporting practices. All items selected by at least one reviewer were retrieved. If more than one publication described a single study and reported different data, they were all included but counted as a single study. AC and ARG, and then CK and ARG met on multiple occasions to jointly review full text articles and resolve eligibility issues. Screening results from the initial search and the updated search were blended to create a single PRISMA diagram.

### Data extraction

A data extraction form was developed to collect information on author, publication year, country, setting, health care issue requiring an intervention, study type (all or partial IM steps), stakeholder involvement, characteristics of the intervention that was designed using the IM process, and any outcomes reported by the study reflecting the impact of the intervention. Details about the intervention were based on the Workgroup for Intervention Development and Evaluation Research (WIDER) reporting checklist and included content, mode of delivery, duration and/or frequency, participants and personnel [16]. For the initial search results, AC and ARG independently pilot-tested the form on the same three articles and compared findings by discussion through four iterations at which time data extraction was congruent, after which AC extracted data from remaining articles. After the search was updated, CK and ARG pilot-tested data extraction on the same three articles and compared findings by discussion; this process was repeated two more times to achieve congruent data extraction, after which CK extracted data from remaining articles. All extracted data were independently checked by UM, who met with ARG on multiple occasions to discuss and resolve data extraction issues.

### Data analysis

Summary statistics were used to describe the number of studies by country, year of publication, study design and health care issue, number of studies using the full or partial IM process, key processes employed in IM steps, intervention design (single or multifaceted), target population for the intervention (patient only, clinician only, patient and clinician), number of studies that employed theory, the most commonly used theories, number of studies involving different stakeholders in co-generation. The most commonly utilized theories, and intervention characteristics and outcomes. The common denominator used for all computations was based on 60 studies representing 61 articles.

## Results

### Search results

**Fig 1:**
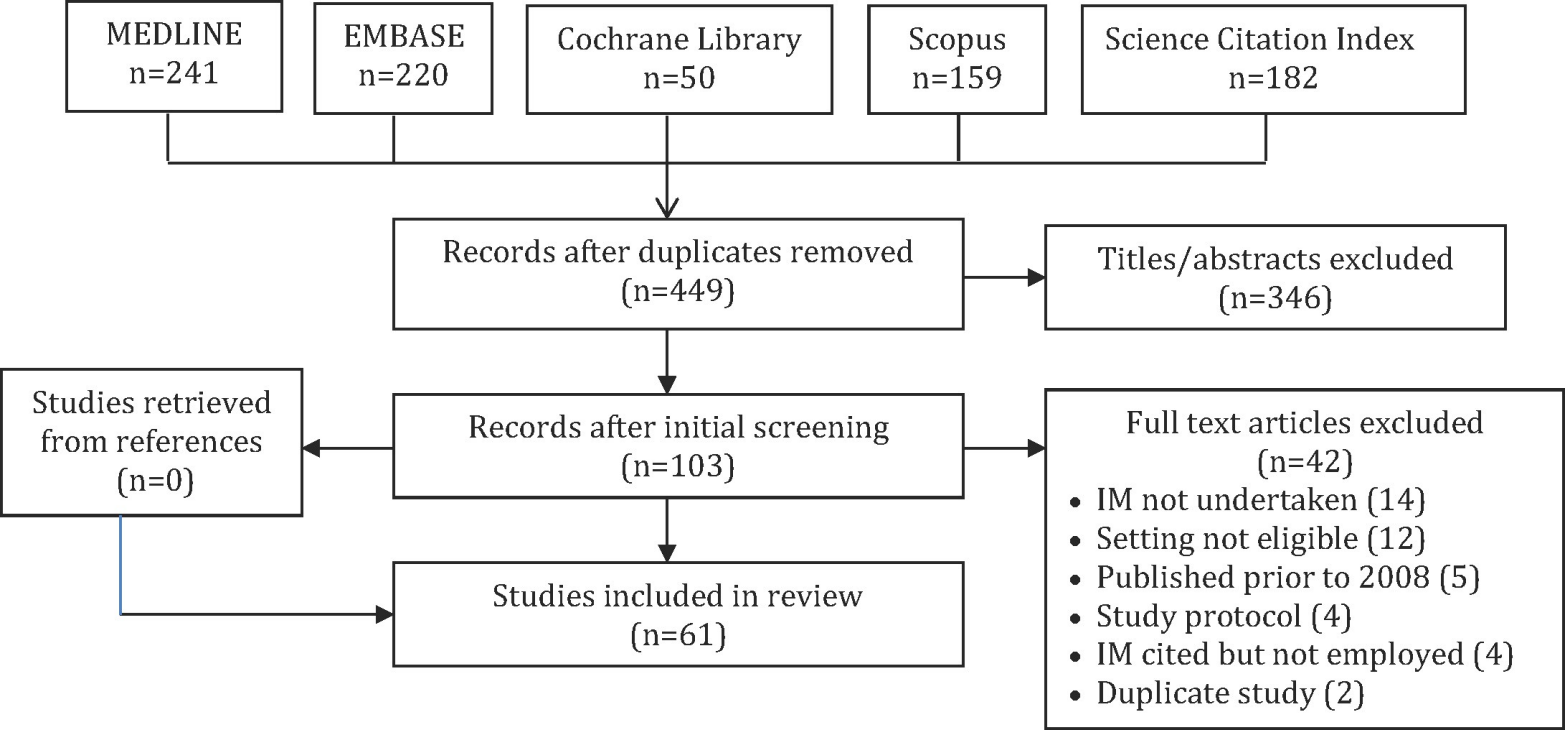
PRISMA diagram.

After duplicate titles were removed, a total of 449 unique titles and abstracts were screened, of which 346 were discarded, leaving 103 full-text articles to be screened. Among those, 42 were excluded: IM not undertaken (14), setting not eligible (12), published prior to 2008 (5), study protocol (4), IM mentioned but not employed (4), and duplicate study (2). Review of references for eligible items yielded no further studies. A total of 61 articles representing 60 studies were included in the review (Fig 1). The data extracted from each study is summarized in S3 Table [17–78].

### Study characteristics

**Fig 2:**
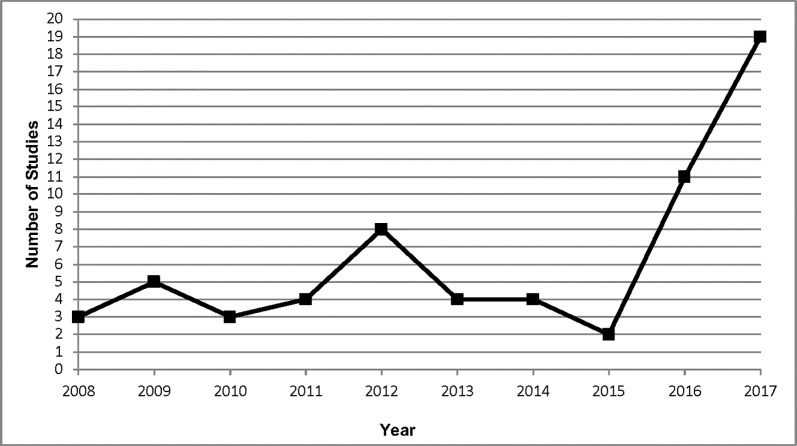
Number of IM studies published per year.

Included articles were published from 2008 to 2017 inclusive. While not continuous, the number of studies published per year increased from a high of 8 in 2012 to 19 in 2017 (Fig 2). This translates to half of included studies published in the last two years (30/60, 50.0%). Studies were conducted in 13 countries, most often in the Netherlands (22, 36.7%) followed by the United States (16, 26.7%), Canada (8, 13.3%), United Kingdom (6, 10.0%) and Iran (2, 3.3%). One study (1.7%) was conducted in each of Australia, Brazil, Denmark, Estonia, Germany, Italy, South Africa and South Korea.

Table 1 summarizes the disease or health service modalities addressed in each study. The most common disease or health service modality categories were health promotion (15, 24.2%), preventive medicine (11, 17.7%), cardiovascular disease (10, 16.1%), and mental health (8, 12.9%).

**Table 1:** Number of studies by disease or health service modality.

Disease Category^a^	Studies n (%)
Preventive medicine	10 (16.7)
Mental health	7 (11.7)
Cardiovascular disease	10 (16.7)
Health promotion	15 (25.0)
HIV-AIDS	5 (8.3)
Genitourinary disorders	2 (3.3)
Immunologic conditions	3 (5.0)
Cancer	5 (8.3)
Digestive system disorders	3 (5.0)

^a^Based on ICD-10 version 2016 (apps.who.int/classifications/icd10/browse/2016/en)

With respect to research design, the majority of studies employed multiple methods including one or more approaches for Needs Assessment plus one or more approaches for Evaluation of a program or intervention generated through the IM process (46, 76.7%). For the 14 (23.3%) of studies employing a single research design, 4 (23.5%) were randomized controlled trials, 3 were survey studies, 2 studies used focus groups, 2 studies used interviews, 2 studies employed a prospective observational research design, and 1 study involved a literature review.

### IM steps employed

Table 2 summarizes the IM steps used by each included study. Among included studies, 73.3% (44/60) employed all 6 IM steps. The remaining studies employed steps 1 to 4 (10, 16.7%), step 1 only (3, 5.0%), steps 2 to 6 (2, 3.3%) and steps 1 to 3 (1, 1.7%).

**Table 2:** Intervention Mapping steps used by included studies.

Study	Intervention Mapping Steps						
	1. Conduct needs assessment	2. Set program objectives	3. Select theory-based methods	4. Develop intervention plan	5. Develop implementation plan	6. Develop evaluation plan	Steps employed
Athilingam 2017 [17]	x	x	x	x	x	x	6
Besharati 2017 [18]	x	x	x	x	x	x	6
Caminiti 2017 [19]	x	x	x	x	—	—	1–4
Cho 2017 [20]	x	x	x	x	x	x	6
Cote 2017 [21]	x	x	x	x	x	x	6
DeBate 2017 [22]	x	x	x	x	x	x	6
Dumas 2017 [23]	x	x	x	x	x	x	6
Golsteijn 2017 [24]	x	x	x	x	x	x	6
Krops 2017 [25]	x	x	x	x	—	—	1–4
McEwen 2017 [26]	x	x	x	x	—	—	1–4
Merkx 2017 [27]	x	x	x	x	x	x	6
Miranda 2017 [28]	x	x	x	x	x	x	6
Muir 2017 [29]	x	x	x	x	x	x	6
Puijk-Hekman 2017 [30]	x	x	x	x	x	x	6
Sakakibara 2017 [31]	x	x	x	x	x	x	6
Shegog 2017 [32]	x	x	x	x	x	x	6
van Belle 2017 [33]	x	x	x	x	x	x	6
van Dongen 2017 [34]	x	x	x	x	—	—	1–4
van Dulmen 2017 [35]	x	x	x	x	x	x	6
Beentjes 2016 [36]	x	x	x	x	x	x	6
Dalum 2016 [37]	x	x	x	x	x	x	6
Greaves 2016 [38]	x	x	x	x	x	x	6
Hall 2016 [39]	x	—	—	—	—	—	1
Jones 2016 [40]	x	x	x	x	x	x	6
Kerstenetzky 2016 [41]	x	—	—	—	—	—	1
Norris 2016 [42]	x	x	x	x	x	x	6
Romeike 2016 [43]	x	x	x	x	—	x	6
Shakibazadeh 2016 [44]	—	x	x	x	x	x	2–6
Smith 2016 [45]	x	x	x	x	—	—	1–4
Steffen 2016 [46]	x	x	x	x	—	—	1–4
Gray 2015 [47]	x	—	—	—	—	—	1
Highfield 2015 [48]	x	x	x	x	x	x	6
Cabassa 2014 [49]	x	x	x	x	—	—	1–4
Geidl 2014 [50]	x	x	x	—	—	—	1–3
Hesselink 2014 [51]	x	x	x	x	x	x	6
De Brito-Ashurst 2013 [53]	x	x	x	x	—	—	1–4
Laisaar 2013 [54]	x	x	x	x	x	x	6
Munir 2013 [55]	x	x	x	x	x	x	6
Theunisen 2013 [56]	x	x	x	x	x	x	6
Byrd 2012 [57]	x	x	x	x	x	x	6
Cherrington 2012 [58]	x	x	x	x	x	x	6
Cornelio 2012 [59]	x	x	x	x	x	x	6
Gillison 2012 [60]	x	x	x	x	—	—	6
Noordegraaf 2012 [61]	x	x	x	x	x	x	6
Scarinci 2012 [62]	x	x	x	x	x	x	6
Suzuki 2012 [63]	x	x	x	x	x	x	6
Zwikker 2012 [64]	x	x	x	x	x	x	6
Hanbury 2011 [65]	x	x	x	x	x	x	6
Looijmans-van Akker 2011,2009 [66,67]	x	x	x	x	x	x	6
van Der Veen 2011 [68]	x	x	x	x	x	x	6
van Rijssen 2011 [69]	x	x	x	x	x	x	6
Detaille 2010 [70]	x	x	x	x	x	x	6
Koekkoek 2010 [71]	x	x	x	x	x	x	6
Schmid 2010 [72]	x	x	x	x	x	x	6
Albada 2009 [73]	x	x	x	x	x	x	6
Bartholomew 2009 [74]	—	x	x	x	x	x	2–6
Ducharme 2009 [75]	x	x	x	x	x	x	6
Ramirez-Garcia 2009 [76]	x	x	x	x	x	x	6
Cote 2008 [77]	x	x	x	x	—	—	1–4
Fransen 2008 [78]	x	x	x	x	—	—	1–4

#### Step 1: Needs assessment

All but 2 studies conducted a Needs Assessment (58, 96.7%). Most of the studies that conducted a Needs Assessment employed multiple approaches to assess determinants (facilitators and barriers) of target performance objectives (50, 84.7%). Literature or systematic reviews (49, 98.0%), qualitative interviews or focus groups (43, 86.0%), and questionnaires (24, 48.0%) were commonly used. Other approaches included discussion among stakeholders such as town hall meetings (13, 26.0%), review of documents or medical records (2, 4.0%), and concept mapping (2, 4.0%). Among 8 (13.8%) studies that assessed determinants using a single approach, 4 (50.0%) employed literature reviews, 3 (37.5%) used questionnaires, and 2 (25.0%) used either qualitative interviews or focus groups.

#### Step 2: Program objectives

All but 2 studies (58, 96.7%) explicitly stated Program Objectives including desired behavioural outcomes and change objectives among a specific population in either the form of a matrix or as stated objectives.

#### Step 3: Theoretical methods and practical strategies

A total of 55 (91.7%) studies considered a Theoretical Framework to inform the selection and design of programs or interventions. Among these studies, 37 (67.3%) used more than one theory. Social Cognitive Theory (24, 43.6% of 55), the Health Belief Model (10, 18.2% of 55), and the Theory of Planned Behaviour (10, 18.2% of 55) were named most frequently by these studies.

#### Step 4: Program or intervention design

Of the 56 (93.3%) studies that described Program Design, programs or interventions were pre-/pilot-tested by 34 (60.7%) studies to refine their design. For example, through qualitative interviews and satisfaction questionnaires with 23 patients and 12 caregivers, one study evaluated the acceptability of a heart failure self-help resource that aimed to engage patients and caregivers in exercise and symptom management [38]. This resource was reported acceptable and patients and caregivers were satisfied with the content and format of the resource. In another example, the authors evaluated an educational program that consisted of individual learning sessions for caregivers of patients with Alzheimer’s disease [75]. Using qualitative interviews with two caregivers, the authors found that both caregivers reported acquiring learning from the program, using reframing as a coping strategy, and improved communication with their affected relatives.

Among the 57 studies that described the program/intervention generated through use of the IM process, 40 (70.2%) targeted patients, 8 (14.0%) targeted clinicians, and 9 (15.8%) were aimed at both patients and clinicians. Of the 40 programs/interventions targeting patients, 24 (60.0%) described educational meetings or materials, 23 (57.5%) described social strategies such as clinicians supporting patients with head and neck cancer to determine their rehabilitation goals, actions, and coping plans [26], 8 (20.0%) were self-management apps or websites, and 3 (7.5%) were tools/guides, for example, a pre-chemotherapy online communication tool for patients to communicate with their nurses throughout treatment [35]. Among the 8 programs/interventions targeting clinicians, 6 (75.0%) involved educational materials or meetings and 3 (37.5%) involved point-of-care resources or tools. Of the 9 programs/interventions aimed at both patients and clinicians, 2 (22.2%) involved educational materials or meetings, 2 (22.2%) involved social strategies such as a resistance exercise coaching program for elderly individuals [34], and 2 (22.2%) employed point-of-care tools, for example, a tool to facilitate communication between patients and nurses [33].

#### Step 5: Adoption and implementation

A total of 44 (73.3%) studies addressed Adoption/Implementation. Of these studies, 38 (86.4% of 44) mentioned a plan to implement the program or intervention. Of those, 24 (54.5%) studies offer non-specific or vague details. For example, expert groups were asked for feedback on program design and implementation [30], or an implementation guide was developed but no details were provided [57]. Remaining studies that mentioned an implementation plan (20, 45.5%) provided somewhat greater detail about who would implement the program or intervention and how. For example, details were provided about who generated an implementation plan for a 45-minute mental health counselling session and how the plan was devised, and details were provided on the implementation strategy, which comprised a 3-day training program for all clinicians, after which sessions were supervised by the first author with hands-on support as well as email and telephone support [71].

#### Step 6: Monitoring and evaluation

Among 43 (71.2%) studies that addressed Monitoring/Evaluation, 20 (46.5%) provided rudimentary details about how the program or intervention would be evaluated in future, 19 (44.2%) described how the intervention was in the process of being evaluated but did not report the results, and 4 (9.3%) reported the impact or outcomes of program or intervention evaluation.

Of the 4 studies that reported the impact or outcomes of the program or intervention generated through use of the IM process, 3 reported positive findings [18,22,74] and 1 [65] reported mixed results. All 3 studies that reported positive findings reported all 6 IM steps [18,22,74]. The single study that achieved mixed results reported IM steps 2 to 6, but was published in 2009 before IM step 1, needs assessment, was added to the IM process [65]. The effectiveness of an educational intervention to increase colorectal cancer screening rates was evaluated using a randomized controlled trial in four groups of participants (men and women) from eight health centres [18]. Four months after the intervention, screening rates increased significantly to 87.1%, 61.3%, 54.8% and 1.6% for participants in the following intervention conditions: education and free Fetal Occult Blood Test (FOBT) screening, online education, free FOBT screening only, and control group, respectively (p<0.001). A randomized controlled trial was used to determine that an eating disorder prevention educational program and materials significantly improved knowledge about the causes and treatments of eating disorders among patients and health care providers (p<0.001) in 27 dental and dental hygiene training programs [22]. A pre- and post-questionnaire study found significantly improved expectations for positive patient outcomes and intention to prescribe diuretics among 147 health care educators following an educational program that comprised of in-person workshops that supported clinician educators to disseminate the results of a blood pressure treatment guideline [74]. In the fourth study, which reported conflicting results, a mixed methods design was used to evaluate an educational meeting that aimed to increase clinician adherence to a suicide prevention guideline [65]. Statistical modeling showed that the intervention did not have a statistically significant impact on adherence to the guideline (p>0.05, R^2^ = 0.27). A survey of health professionals showed that, apart from perceived behavioural control (p<0.027), there was no change in scores for attitude (p = 0.80), subjective norm (p = 0.76) or intention to use the guideline (p = 0.84).

### Stakeholder involvement

S3 Table summarizes if and how stakeholders were engaged in the IM process. Among 60 included studies, 17 (28.3%) made no mention of involving stakeholders in any IM steps reported. Among the 43 (71.7%) studies that did mention stakeholder involvement, all but two (41, 95.3%) specified the types of stakeholders engaged. Most studies involved a combination of the following stakeholder groups: patients, clinicians, community organizations or representatives, and researchers; 12 (29.3% of 41) involved clinicians only, 5 (12.2% of 41) involved patients only, and 13 (31.7% of 41) involved both clinicians and patients. Community organizations and representatives were involved in 10 (24.4%) of the included studies.

Of the 43 studies that mentioned stakeholder involvement, less than half (21, 48.8%) described how they were engaged. In those 21 studies, stakeholders were members of committees or planning groups. The frequency of meetings was mentioned in 6 (28.6%) of the 21 studies and varied from quarterly, monthly or weekly, or a finite number of meetings held during the process. Four (19.0%) studies involved stakeholders on committees or groups in discussing the findings of Needs Assessment (Step 1). All 21 studies involved stakeholders in Program Design (Step 4) such as the review of program/intervention content and/or format (8, 38.1%), creation of program/intervention materials (7, 33.3%), recruitment of participants (6, 28.6%), and tailoring the program/intervention to the target population (3, 14.3%). Three (14.3% of 21) studies engaged stakeholders in developing program objectives and matrices of change outcomes. One (4.8%) study involved stakeholders in selecting the theory-based methods and strategies to guide the IM process. Two (9.5%) studies solicited stakeholder input and guidance on developing the intervention plan. Finally, 1 (4.8%) study involved stakeholders in reviewing the evaluation plan of intervention.

## Discussion

The use of IM has steadily increased since 2008 and doubled from 2016 to 2017. IM was used in 13 countries to plan single or multi-faceted programs or interventions, which were largely based on educational materials or meetings aimed at patients, clinicians or both, to improve health care delivery or associated outcomes for a wide variety of clinical issues or conditions. Most studies reported having completed all 6 IM steps, and nearly all studies completed at least the first 4 IM steps, meaning that a program or intervention was developed. Activities undertaken for the first 4 steps, Needs Assessment, Program Objectives, Theoretical Framework and Program/Intervention Design, were well-reported by nearly all studies. While many studies reported how they planned to implement the program or intervention in future (step 5), fewer reported pilot-testing the program or intervention (part of step 4), or provided details about current or planned evaluation (step 6). Of only 4 studies that evaluated the program or intervention, 3 reported improvements in patient or clinician knowledge or behaviour. With respect to stakeholder engagement, 72% of studies said that patients, clinicians and/or representatives of community organizations were involved, but few studies reported the activities in which they were engaged or the impact of their engagement.

Two other studies reviewed the use of IM. Garba and Gadanya examined 22 IM studies published from 1999 to 2014 focused on designing disease prevention interventions but largely focused on whether interventions achieved the desired improvements when rigorously evaluated, rather than examining how the IM process was employed [79]. Their review found that IM resulted in significant uptake of disease prevention programs that were specifically evaluated using randomized controlled trials. Durks et al. examined how IM was used in 17 studies published up to May 2015 but focused only on the first 4 IM steps and largely described the health care practices that needed improvement and the proposed interventions [80]. Two additional reviews specific to particular disciplines examined the fidelity of the IM process and described interventions developed using IM. Similar to our review, both studies found that details of stakeholder engagement were sparse, and few interventions achieved the desired improvements. Fassier et al. conducted a systematic review of interventions developed with IM in work disability prevention from 2007 to August 2017 [81]. Among 8 studies included, stakeholder engagement was poorly described, and 2 interventions were reported as effective. Lamort-Bouché et al. conducted a systematic review of interventions developed with IM in the field of cancer up to August 2017 [82]. Stakeholder engagement was described in 6 of 16 studies, and 5 interventions were reported as effective. In contrast to these studies, our review is the most up-to-date, comprehensive of various health care issues or conditions, inclusive of all 6 IM steps, and descriptive of how stakeholders were engaged. We also included many more studies than prior reviews. This may in part be due to the fact that ours is the most recent and the number of IM studies doubled in the last two years of our review (2016, 2017). It may also be due to how we searched for studies; prior reviews employed search strategies, for which it is challenging to achieve an optimal balance of sensitivity and specificity, whereas we searched for any studies that included the phrase “intervention mapping” or cited the original publications that introduced the IM process [12,13], which may have been a more comprehensive approach.

The strengths of this study include use of rigorous scoping review methods [14] and compliance with standards for the conduct and reporting of reviews [15]. Several issues may limit the interpretation and application of the findings. Although scoping reviews often include consultation with stakeholders to interpret the findings, this was not done due to logistics and feasibility. Our search was limited to English language studies, and we did not search the grey literature given the methodological challenges of doing so that have been identified by others [83,84]; therefore, we may not have included all relevant studies. However, as noted earlier, our search approach resulted in a greater yield than previously published reviews of IM [79–82]. We did not search for other studies published by the same authors in an attempt to capture subsequent studies that may have evaluated the impact of the programs or intervention; however, few of the included studies reported evaluation results, and assessing the impact or outcomes of programs or interventions generated by IM was not our goal.

Several implications emerge in relation to the aims of this review, and inform issues that warrant ongoing research. The first aim was to gain insight on how to optimize IM. Overall, IM appears to be largely well-used and reported given the fidelity of details pertinent to its 6 steps. However, it is unclear if the use of IM consistently results in a successful program or intervention because few studies reported evaluation results. To be clear, step 6 of the IM process requires only that an evaluation plan be established, not actually operationalized. While some studies did describe ongoing or future evaluations, we included studies from 2008 to current and expected evaluations would have been published for earlier studies. This is confirmed by prior reviews, which also found that either interventions were not evaluated or they did not achieve improvements [81,82]. Reasons for the lack of evaluation or of effectiveness of programs or interventions generated using IM are unclear. They may have never undergone evaluation, or faced publication bias if the results of evaluation were negative. Given the sharp increase in IM use over the last two years, a future review is warranted to examine whether evaluations of programs or interventions developed more recently get published, and if those programs or innovations, informed by the engaged stakeholders, improve behaviour or outcomes. If effectiveness of programs or innovations developed using the IM process is still not established, then future research could seek to modify IM steps in an effort to optimize the process. That research should first assess whether lack of impact is due to attributes of the program or innovation or the way it was implemented, which could reflect limitations in how stakeholders were involved in the IM process, or barriers or challenges in the environment that prevent the program or innovation from being adopted, factors not related to the IM process that would necessitate other types of interventions that support program/innovation use.

The second aim of this research was to gain insight on how to optimize IKT for IM. Stakeholder engagement in IM is meant to increase the relevance, feasibility and ultimate success of programs or interventions. However, this review found that many studies did not specify if and how stakeholders were involved, or how involvement influenced program or intervention design, and those that did engage patients, clinicians and/or representatives of community agencies provided sparse details. It is unclear how stakeholder characteristics or involvement influenced the design, implementation or evaluation of the program or intervention. Thus, an understanding of how stakeholders should be chosen and involved in IM remains unknown. Although literature on IKT [9] and on patient/public engagement in health service planning and improvement is growing [85], optimal ways to choose and involve various stakeholders that are representative of the target users in research or in health service planning or improvement is limited. In our review, studies that described engagement were most likely to involve stakeholders via committees or planning groups in program design (step 4) by reviewing program/intervention content and/or format. This suggests that stakeholders provided input on already-established programs or interventions, rather than being involved from the outset, which may have resulted in a less-than-ideal program or intervention. If programs or interventions were not tailored based on stakeholder values and context, which has been shown to improve the likelihood of improved behaviour or outcomes [4], or if engagement was tokenistic, this could explain why programs or interventions developed using IM have not achieved the intended impact (previous refs). Future research should investigate the short-term impact of various ways to engage stakeholders on the design of programs or interventions, and the long-term impact of engagement options on the effectiveness of programs or interventions.

## Conclusions

While IM is increasingly widely used many studies did not engage stakeholders, failed to describe how stakeholders were engaged, or involved stakeholders in a tokenistic manner, and many studies did not pilot-test or evaluate the impact or outcomes of the program or intervention. Therefore, the design of programs or interventions may be suboptimal because they were not informed by and tailored to address stakeholder context, and it is unclear if the use of IM consistently results in effective programs or interventions. Those employing IM should more thoroughly report how stakeholders were involved in each IM step and how involvement influenced program or intervention design. They should also report details of ongoing or planned evaluation, or specify if no such evaluation is planned and the rationale. Future research should investigate how to optimize stakeholder engagement in IM, and whether use of IM itself or stakeholder engagement in IM are positively associated with effective programs or interventions.

## Supporting information

S1 Table PRISMA checklist. (DOCX)

S2 Table Intervention Mapping process. (DOCX)

S3 Table Data extracted from included studies. (DOCX)
