# Supplementary material for: Engaging stakeholders in the co-development of programs or interventions using Intervention Mapping: A scoping review
Source: PLoS One. 2018 Dec 26;13(12):e0209826. doi: 10.1371/journal.pone.0209826 (PMC6306258; doi:10.1371/journal.pone.0209826)
Supplement: S2 Table — (DOCX) [file pone.0209826.s002.docx]

S2 Table. Intervention Mapping process [12,13]

| Steps | Components | Definition |
| --- | --- | --- |
| Needs assessment | Specify or identify the determinants | Assess determinants of the stated performance objectives using methods such as literature review, surveys, or qualitative interviews or focus groups |
| Program Objectives  *Identifies who and what will change* | Identify desired behaviour and outcomes | Reflect on what participants need to perform the health-related behaviour |
|  | Differentiate target population | Consider sub-groups defined by factors such as diagnosis/stage, other physiological factors, age, SES, ethnicity/literacy or other factors |
|  | Develop a matrix of program objectives | Develop one or more matrices (i.e. patient, provider, organizational, system level) of behaviour by determinants. Each cell specifies learning objectives and change objectives. |
| Theoretical Methods & Practical Strategies  *Matches interventions to program objectives* | Brainstorming methods | Establish a series of questions in the format: How can change in the determinant be influenced to achieve program objectives |
|  | Delineating methods | Assemble a provisional list of theoretically or empirically-derived interventions based on effectiveness |
|  | Translating methods into strategies | Assess the provisional interventions for relevance and feasibility |
| Design the Program  *Develop and test prototype* | Operationalize the strategies | Specify scope, sequence, content, format, mode and delivery of program strategies/components |
|  | Design program materials | Develop strategies/components and accompanying instructional or training material |
|  | Pre-test program materials | Assess reaction, satisfaction, acceptability, clarity, etc. |
| Adoption & Implementation  *Generate a detailed plan of what needs to be done to ensure program delivery* | Develop a linkage system | Engage program users and implementers |
|  | Identify measures of implementation | Design an implementation plan along with measures of adoption and implementation |
|  | Specify determinants of adoption and implementation | Identify barriers and enablers of adoption and implementation |
|  | Write a plan of implementation | Detail the methods and strategies used to promote dissemination, adoption, implementation and maintenance of the program |
| Monitoring & Evaluation  Generate tools for evaluation | Develop an evaluation model | Performance objectives provide a basis for measuring behaviour and environmental outcomes; determinants identify constructs to be measured, and change objectives provide content items to form measurement scales. Time frames are specified within which change should occur. |
|  | Measure process and effect | Use the evaluation kit to assess program impact and outcomes |
