# Supplementary material for: Engaging stakeholders in the co-development of programs or interventions using Intervention Mapping: A scoping review
Source: PLoS One. 2018 Dec 26;13(12):e0209826. doi: 10.1371/journal.pone.0209826 (PMC6306258; doi:10.1371/journal.pone.0209826)
Supplement: S3 Table — (DOCX) [file pone.0209826.s003.docx]

S3 Table. Data extracted from included studies

| **Study**  [author, year, country, disease, research design] | **IM process** | **Stakeholder involvement** | **Intervention** | **Evaluation** |
| --- | --- | --- | --- | --- |
| Athilingam  2017 [17]  United States  Heart failure  Multiple (quantitative survey, qualitative interviews) | Needs assessment  Literature review on heart failure self-care needs, mobile phone use in the United States, and current mobile phone-based interventions for chronic disease and heart failure; interviewed 10 heart failure patients and 4 cardiologists.  Feasibility study with 37 participants assessed usability, ease of use, accuracy of material, problem solving, and patient satisfaction, but methods NR.  Program objectives  Proximal/primary outcome was increase in patient engagement with HeartMapp; distal/secondary outcome was improved self-care routine.  Theoretical framework  Bandura’s Self-Efficacy Theory from the Social Cognitive Theory; Information, Motivation, Behavioural Skills (IMB) Model; and Carmen’s Multidimensional Model of Patient Engagement.  Designing the Program  Rounds of alpha and beta testing were conducted to pilot test and assess feasibility of the intervention.  Implementation Plan  NR  Evaluation Plan  HeartMapp will be developed for iOS and Windows mobile platforms and translated to Spanish before the intervention is evaluated. | NR | Patients  Single  HeartMapp mobile phone application to offer tailored alerts and feedback on weight, blood pressure, heart failure symptoms, exercise, medical list and scheduling medication use, vital signs monitoring, and heart failure information. | NR |
| Besharati  2017 [18]  Iran  Colorectal Cancer  Multiple (quantitative survey, qualitative interviews, qualitative focus groups, randomized trial) | Needs assessment  Needs assessment included a literature review (recognize health problem and determinants of colorectal cancer screening in asymptomatic individuals); ten focus groups with men and women who have not undergone colorectal cancer screening; 20 in-depth interviews with men and women who have undergone screening tests; 12 in-depth interviews with physicians; and a questionnaire survey, designed from literature review and qualitative data, that was piloted in 30 adults and then used to gather data from 480 adults.  Program objectives  Two program objectives: (1) to increase colorectal cancer screening (FOBT) among adults, and (2) increase in referral for colorectal cancer screening.  Theoretical framework  Health Belief Model (HBM); Theory of Planned Behaviour (TPB); Social Cognitive Theory (SCT); and Preventive Health Model (PHM) was used as an integrated model.  Designing the Program  Based on needs assessment data and additional focus group discussion held with target group for collecting information about preferences for intervention tools, the planning team developed a list of intervention materials and activities.  The intervention was piloted in two health centres in January 2016. For program implementation, 248 adults were targeted, the target group was divided into 4 groups and 31 adults were included at each health center (8 health centers total). Adults were also asked to read the pamphlet at the end of the pilot intervention.  Implementation planning  Participation of adults in choosing appropriate strategies and materials for implementing the intervention; pretesting the materials.  Evaluation planning  Process measures were based on questionnaires for target group and checklist for educator. The process measures were: participation rate, program fidelity, dose and satisfaction of program. Program effectiveness was measured through a randomized controlled trial. | NR | Patients  Single  Two sessions at health centres during a two-week period with reminder pack containing postcards and pamphlet, and an educational video titled “Being a winner in life: how to prevent colorectal cancer cancer.”  The first session involved an educator speaking about colorectal cancer and screening recommendations. In the second session, the video was shown, and a role-playing activity was simulated.  Two months after the training sessions, participants received a reminder phone call that encouraged them to undergo FOBT screening. | The preliminary evaluation findings revealed that during the 4-month follow up period, colorectal cancer screening rates were 87.1%, 61.3%, 54.8% and 1.6% for participants assigned to education with free FOBT screening, online education, only free FOBT screening and control group, respectively.  Results of pilot intervention indicated that >90% of participants were satisfied with: intervention content, schedule time, and the number of training sessions. Adults also indicated that the pamphlets were understandable and interested, and preferred black text on white background and clearer pictures. |
| Caminiti  2017 [19]  Italy  Stroke  Multiple (quantitative survey, qualitative focus groups) | Needs assessment  Conducted a population and an in-hospital survey, aiming to measure stroke awareness and preparedness and to analyze the behavioural determinants of prehospital delay defined as patient admission later than 2 hours after symptom onset. Between February and March 2013, 202 population surveys were collected at the 12 community-based sites.  393 patients were assessed for median time from symptom onset/awareness to presentation at the hospital.  Program objectives  Low stroke recognition, not immediately referring to emergency medical services or scarce knowledge about treatment opportunities in the first few hours are determinants of prehospital delay.  Theoretical framework  General Model of Total Patient Delay, Common Sense Model (CSM) of self-regulation, and definition of narrative communication.  Designing the Program  Based on needs assessment data and additional focus group discussion held with target group for collecting information about preferences for intervention tools, the planning team developed a list of intervention materials and activities.  Between February and March 2013, 202 questionnaires were collected at the 12 community-based sites.  Five focus groups (total 35 participants) conducted between March 2013 and October 2013 to test educational products and identify the most suitable site to disseminate the message. Each focus group had five sessions: signing informed consent, discussion on participants’ experiences with stroke, presentation of the educational product prototypes, exchanging views on the prototypes, and identification of public places and public events deemed suitable for campaign message dissemination. The reliability of the message, its specificity to stroke, the use of narrative method, and of cartoon, the layout, clarity, and efficacy of key messages were evaluated by participants on a 10-point Likert scale.  Implementation planning  NR  Evaluation planning  NR | A steering committee consisting of: Principal Investigator, three  referring clinicians for the participating centers, one  communication expert, one methodologist, one psychologist,  one representative of ALICe, one emergency medical service physician,  one journalist, and the representative of the communication  agency in charge of campaign organization.  The committee met six times over a period of 3 months and applied the following IM steps: developing matrices of change, selecting theory-based methods and strategies, and designing and organizing the program. | Patients + clinicians  Multifaceted  Campaign was developed that included a comic strip, one poster, and twos animation videos.  Campaign strategies included: mail delivery of brochures, displaying brochures in public spaces, broadcasting animation videos in hospital waiting rooms and TV stations, and setting up booths to distribute campaign material. | The choice of the family setting was deemed appropriate in the light of data obtained from the patient survey; 77% of respondents did not live alone, and 73% was over 65 years old. Also, the family proved to be the most important resource of promotion of correct behavior: 43% of individuals who arrived at hospital within 2 hours had been advised by a family member to call EMS. |
| Cho  2017 [20]  South Korea  Walking  Quantitative survey | Needs assessment  A literature review on the influence of determinants for physical activity as well as a focus on effective community-based interventions of exercise programs. A survey with 21 Korean-Chinese migrant women collected information about their needs for enhancing cultural adaptability and for assessing the degree of mobile phone use as a possible means of intervention delivery.  Five Korean-Chinese migrant women participating in the walking exercise for three weeks, after which they discussed in a group their experiences of the walking exercise, the primary places where they did the walking exercise, and suggestions to enhance the walking exercise.  Program objectives  (1) Regular walking exercise above a moderate level; and (2) induce change in sociopsychological and cognitive capabilities (improvement of self-efficacy, increase of social support, enhancement of health-related knowledge, and enhancement of acculturation level).  Theoretical framework  The Interaction Model of Client Health Behaviour (IMCHB); and Social Cognitive Theory.  Designing the Program  Creation of an intervention manual to promote fidelity in intervention delivery. Program materials were developed, feedback from Korean-Chinese migrant community was elicited, and the feasibility of implementing the program in migrant community centers was assessed.  Implementation planning  Marketing the program: distribution of recruitment posters and culturally-adapted progress newsletters.  Five individuals were recruited from the target population to contribute to the design and evaluation aspects of the implementation.  Evaluation planning  Self-reported surveys, daily diary, and objective measures for health outcomes such as blood pressure, glucose level, cholesterol level, and body mass index. | Steering Committee: Six leaders from local society organizations, including the person who is responsible for the foreign workers support center.  Committee meetings occurred twice, and the focus was to identify the characteristics of female Korean-Chinese workers.  Five individuals from target population were commissioned as health leaders and contributed to the development of the program and creation of study tools, educational materials, acculturation content, and recruitment of participants, and served as facilitators and supporters of the program. | Patients  Multifaceted  (1) Setting up a personalized exercise goal to enhance self-efficacy and a consultation to overcome various obstacles; (2) provisioning of acculturation content design to increase social support and enhance cultural adaptation; (3) development and provision of health education programs and educational materials to enhance health knowledge.  Exercise intervention with a 10,000 step walking goal and stretching before and after walking. Reinforced sessions: goal setting session, individual consultation, and acculturation content. | NR |
| Cote  2017 [21]  Canada  HIV  Multiple (qualitative focus groups, randomized trial) | Needs assessment  Literature review of lifestyle risk factors and predictors of healthy behaviours.  Program objectives  To support people living with HIV in the adopt of healthy behaviours.  Theoretical framework  Theory of Planned Behaviour.  Designing the Program  A focus group was conducted with people who live with HIV/AIDS (n = 6) to inform the development of the intervention content.  The intervention content has been validated by different health professionals, including a nutritionist, a kinesiologist, nurses, and physical activity research experts.  Implementation planning  NR  Evaluation planning  An online randomized controlled trial with parallel groups is currently ongoing across Canada with a convenience sample of participants (n = 750). | Researchers from different disciplines (nursing, medicine, social work, health  promotion, and computer science). | Patients  Multifaceted  *TAVIE en santé,* a tricomponent web-based tailored intervention facilitated by a virtual nurse and consists of seven interactive 5-10 minute-web sessions for smoking cessation, physical activity, and healthy eating. | NR |
| DeBate  2017 [22]  USA  Eating Disorders and dental hygiene  Randomized trial | Needs assessment  A literature review and collected information from dental school deans and dental hygiene program directors for a study published in 2007.  Program objectives Overall Goal: Improve the quality of life of those with eating disorders.  Health provider objectives:  a) Assess patients for signs of an eating disorder; b) Communicate the link between eating disorders and oral health; c) provide patient-specific oral health treatment plans; d) arrange a more frequent recall for patients exhibiting signs of an eating disorder; e) refer patients who exhibit intra- and extra-oral signs of an eating disorder for physical and mental health treatment; and f) communicate with the patient’s primary health care provider.  Patient objectives:  a) Increase adherence to patient-specific oral health treatment plans; b) increase physical and mental health care; and c) decrease engagement in eating disorder behaviors.  Theoretical framework  Information Motivation Behavioral Skills (IMB) Theoretical Framework; Health Belief Model (HBM); and Precaution Adoption Process Model.  Designing the Program  Intervention development was guided by Brief Motivational Interviewing concepts and processes, active-learning training strategies, and web-based methods  Implementation planning  NR  Evaluation planning  Employing the RE-AIM evaluation framework, the authors conducted a prospective group-randomized controlled trial with dental and dental hygiene classes (n = 27) from accredited programs (n = 12) to assess the efficacy of the EAT program on knowledge, motivation, skills, and self-efficacy of secondary prevention of eating disorders.  The web-based training prototype was tested for value of content and consumer satisfaction in a sample of oral health care providers and students (n = 66). A web-based 11-item Likert scale instrument was used to assess the impact of the program. | NR | Patients  Multifaceted  Oral health e-curriculum titled “Evaluating, Assessing, and Treating Sensitive Oral Health Issues: Case Studies on Oral Manifestations of Eating Disorders” (EAT).  Consists of a didactic component (describes the main types of eating disorders and associated characteristics and health issue); behavioural skills component (EAT Framework and Skills and Interactive Video Case Studies).  Resources include printer-friendly education materials, patient-specific treatment plan templates, and referral to two treatment networks  (mental health and nutrition). | Statistically significant changes from pre- to post-intervention: knowledge of oral manifestations from restrictive eating disorder behaviours and purging eating disorder behaviors; knowledge of treatment options for patients suspect of engaging in eating disorder behaviors; self-efficacy; and role beliefs pertaining to secondary prevention of eating disorders including sub-constructs professional responsibility and liability. Effect sizes for all outcomes ranges from r = 0.62 to 0.83. |
| Dumas  2017 [23]  Canada  Healthy Eating  Multiple (qualitative focus groups, randomized trial) | Needs assessment  Literature search on theoretical domains predicting Vegetables and Fruits intakes and Milk and Alternatives intakes in adults (i.e., knowledge, beliefs about capabilities, beliefs about consequences, intention/goals); and a qualitative study including focus groups to identify female (n = 33 French-Canadian women) Internet users’ perceptions of their use of healthy eating blogs.  Program objectives  Two behavioral outcomes were selected: (1) in the next six months, mothers will increase their consumption of Vegetables and Fruits to include seven servings per day, and (2) in the next six months, mothers will increase their consumption of Milk and Alternatives to include two servings per day.  Theoretical framework  IM taxonomy; 93-item Behavior Change Technique Taxonomy v1; Theoretical Domains Framework; and Motivational Interviewing (MI).  Designing the Program  Focus group results guided the sequence and design of blog and writing the blog posts. A 6-month intervention was developed aimed to address one performance objective per month in weekly blog publications written by a registered dietitian with a total of 26 weekly posts will be published.  The blog was created on WordPress and included the following components: home page, identification of the blogger, archiving system of blog posts for navigation, blog posts divided by subtitles and paragraphs to facilitate reading, internal and external links to references to scientific papers or other reputable sources, and a comments section for communication between blogger and study participants. The blog also consisted of a Recipe page based on categories of meals and a Resource page with supplementary tools and guides.  Implementation planning  Two authors will give an orientation about how to use the blog to all study participants. Women who have not logged onto the blog for at least two weeks will be reminded by email.  Sought to include engagement-promoting methods (e.g., peer and counselor support) to promote mothers’ use of the blog and adherence to the intervention.  Evaluation planning  Randomized controlled trial has been launched to evaluate the effects of the blog on dietary behaviors of French-Canadian mothers. This trial will recruit 110 mothers. Recipes were pretested by two authors and their families. | Blogger (registered dietician who wrote the blog posts), and website designer. | Patients  Single  Weekly blog publications  (1) mothers have Vegetables and Fruits and/or Milk and Alternatives with every meal, (2) mothers plan adequately Vegetables and Fruits and Milk and Alternatives purchases and meal preparation, (3) mothers make healthy food choices at the grocery store, (4) mothers know economic food options to increase daily intake of Vegetables and Fruits and Milk Alternatives, (5) mothers increase the daily intake of Vegetables and Fruits and Milk and Alternatives of the family, and (6) mothers make healthy substitutions in recipes. | NR |
| Golsteijn  2017 [24]  Netherlands  Prostate and colorectal cancer  Multiple (qualitative interviews, randomized trial) | Needs assessment  Systematic literature search about motivating and hindering factors of cancer prostate and colorectal survivors’ regarding their behaviour and preferences in a physical activity program. To confirm and expand this information, the authors conducted interviews with our target group n = 29) and healthcare professionals (n = 15) about physical activity advantages, cancer-specific barriers to physical activity and information and intervention preferences regarding a computer-tailored intervention among our target group.  Program objectives  The main goal of the OncoActive intervention is to increase and maintain physical activity behavior of prostate and colorectal cancer patients/survivors.  Theoretical framework  Social Cognitive Theory.  Designing the Program  The intervention is based on behavior change techniques and aimed at increasing awareness of physical activity behavior and stimulating physical activity during leisure time and in daily activities.  Intervention texts were edited and shortened by a professional editor. Texts and information for using the pedometer for monitoring and goal-setting was developed. To create videos for participants, cancer survivors were interviewed and video-taped. A module was also designed to help participants communicate with physical therapists about physical activity. FAQs were developed for participants and continuously updated throughout the program.  The intervention was delivered both in-print and online.  Authors evaluated two possible designs for the intervention website using a Likert scale questionnaire. Also pretested the safety and feasibility of the content with cancer care professionals (n = 11).  Implementation planning  For implementation of the OncoActive intervention, the authors created a network of hospitals and radiotherapy institutes in the Netherlands, including the two who participated in the small-scale pilot. Contact persons within these institutions were surgeons, oncologists, urologists, research nurses and nurse practitioners. Daily and weekly regional newspapers, relevant websites and discussion groups were contacted to publish a call for cancer survivors.  Evaluation planning  For this evaluation the authors used a randomized controlled trial design (sample NR) to compare the intervention group receiving the OncoActive intervention (who had also access to all usual care) to the usual care-only control group. | Contact persons within these institutions were surgeons, oncologists, urologists, research nurses and nurse practitioners. They support the intervention implementation by providing resources to participants. | Patients  Multifaceted  Intervention participants receive tailored advice at three time points: (1) two weeks after completing the questionnaire, (2) two months after first advice, and (3) three months after completion of first questionnaire. Each participant also received 2-3 news updates about the intervention and other resources.  Computer tailored physical activity program including video content and quotes from real cancer cancers; one module through which a cancer survivor within the intervention could consult with a physical therapist regarding physical activity; module includes a list of FAQs. | NR |
| Krops  2017 [25]  The Netherlands  Physical disability  Qualitative focus groups | Needs assessment  Four semi-structured focus groups with experts (n – 28) working in the field of physical activity for people with a disability.  Program objectives  Stimulate physical activity and physical activity behaviour in people with disabilities.  Theoretical framework  Theory of Planned Behaviour; Physical Activity for People with a Disability (PAD) Model  Designing the Program  Marketing, collaborations between organizations, feedback, coaching trial period, role models.  Implementation planning  NR  Evaluation planning  NR | NR | NR | NR |
| McEwen  2017 [26]  Canada  Head and neck cancer  Multiple (case series, qualitative focus groups) | Needs assessment  The authors used literature review, focus groups, and advisory panel input to establish the rehabilitation needs of survivors.  Program objectives  (1) Determine the priority rehabilitation needs of survivors; (2) set goals and action plans related to the priority rehabilitation needs; and (3) facilitate the implementation of, and necessary adjustments to, action plans to meet goals.  Theoretical framework  Social Cognitive Theory; Determinants of Self-Management; Global Cognitive Strategies.  Designing the Program  Recruited a rehabilitation professional as a leader of the intervention; prepared for a functional evaluation of participants; created a resource compendium of general cancer and HN cancer-specific rehabilitation resources for survivors and health care professionals.  Iterative series of single cases with 9 adult survivors of cancer who had completed active treatment with a curative intent. These individuals participated in an initial assessment. Semi-structured interviews (n = 5) were also conducted. Several changes were made to the rehabilitation planning consultation intervention from this information.  Implementation planning  NR  Evaluation planning  NR | Advisory panel consisting of survivors, cancer rehabilitation professionals (occupational therapy, physiatry, physiotherapy, and speech–language pathology), and health system representatives. The advisory panel was consulted at all key development junctures. | Patients  Single  Rehabilitation Planning Consultation: Determine goals, actions, and coping plans; and a follow-up to review goals, plans, and results, and to make modifications as necessary. The initial consultation involves a face-to-face meeting whereas the follow-up is either face-to-face or telephone consultation.  The initial consultation consists of the following steps: orientation, consultation, goal-setting, introduction of the cognitive strategy, action planning and coping planning, introduction of online resources, and summary.  The follow-up consultation consists of the following steps: reminder, reorientation, checking and re-planning, and summary. | NR |
| Merkx  2017 [27]  The Netherlands  Gestational weight gain (GWG)  Multiple (quantitative survey, qualitative interviews) | Needs assessment  The authors conducted a systematic review and meta-analysis of the literature on existing interventions for achieving a healthy gestational weight gain; analysed the literature on the determinants and correlates of gestational weight gain in pregnant women; conducted a quantitative survey among healthy pregnant woman to investigate the percentages of women reaching healthy gestational weight gain and to assess the relationship between, among other factors, diet and physical activity and reaching a healthy gestational weight gain; and individual interviews with community midwives to identify their behaviours with respect to promoting healthy gestational weight gain and the determinants of their behaviours.  The authors used the information from the interviews and literature reviews to construct the study model and questionnaire for the quantitative, cross-sectional survey to measure midwives’ self-reported behaviours and determinants.  Program objectives  (1) Help healthy pregnant women to stay within the Institute of Medicine guidelines; and (2) get midwives to adequately support the efforts of healthy pregnant women to gain weight within the Institute of Medicine guidelines.  Theoretical framework  The Transtheoretical Model (TTM); Social Cognitive Theory (SCT); the Elaboration Likelihood Model (ELM); and Attitude-Social Influence-Self-Efficacy model.  Designing the Program  The first part of the program consisted of a 45-minute web-based, tailored program. The second part consisted of a monitoring tool through which women were invited to record their weight during their first visit and thereafter after invitations by email weekly.  For midwives, there was training and an information card. Six training sessions were delivered for 60 midwives and 12 assistants. The information card summarized information, arguments for referral to a dietitian, psychologist or physical activity program, and suggestions for questions.  Implementation planning  Consortium midwives functioned as intermediaries between developers and the final users (pregnant women and midwives) during steps I–IV. Building on a relationship of trust with their pregnant clients, midwives collected a broad understanding of their clients.  Evaluation planning  The intervention was evaluated through a non-randomized pre-post intervention study comparing a historic control cohort with an intervention cohort. Consortium midwives recruited 17 community midwives who participated in the study and also recruited pregnant women. The participants completed a questionnaire that contained items on weight, diet, and physical activity. | Several midwives practising in primary care, a dietitian, a physiotherapist, a psychologist who works with young mothers, an employee of the Royal Dutch Organization of Midwives (KNOV), and health educators.  During the needs assessment, the project team met three times, to discuss the research findings, contribute to the decision-making process on the selection of target groups and advise on the outcomes of the behavioural programme. Between the meetings, the authors consulted with individual project members as needed. | Patients  Multifaceted  'Come On!' is an intervention that promotes adequate gestational weight gain, which consists of an online gestational weight gain program for pregnant women. This program had two parts. | NR |
| Miranda  2017 [28]  Canada  HIV  Multiple (qualitative interviews, qualitative focus groups, randomized trial) | Needs assessment  Interviews with HIV-positive men who have sex with men (n = 10) and HIV counsellors (n = 2), and a literature review.  Program objectives  (1) To plan condom uses when having anal intercourse; (2) to negotiate with a partner the use of a condom during anal intercourse; and (3) to choose to avoid anal intercourse without a condom.  Theoretical framework  Theory of Planned Behavior; and Social Cognitive Theory.  Designing the Program  A focus group of HIV-positive men who have sex with me (n = 10) to explore their perspectives on acceptable methods and strategies for a Web-based intervention.  Implementation planning  Generate an algorithm that decides the sequence through with the intervention would be delivered. Based on participants’ responses on a self-efficacy questionnaire and ranked as either high or low self-efficacy.  The authors planned for the implement throughout the IM process. Before the intervention goes live on the Internet, the Web design team and computer programmer will initiate trial runs with it to test its functioning and implementation over the Internet.  Evaluation planning  A pilot randomized controlled trial is planned to examine the feasibility, acceptability, and preliminary efficacy of the intervention. The results of the pilot trial will be used to make modifications to the intervention. After this process, a full randomized controlled trial will be conducted to evaluate the intervention’s effectiveness in the population. | A multidisciplinary team was established to develop the intervention using the IM process. This team included: a behavioral researcher with expertise in adopting, maintaining, and changing health-related behaviors such as condom use, as well as in Web-based intervention design and delivery; two sexologist researchers with a focus on psychosocial and sociocultural factors associated with at-risk or preventive behaviors, particularly in relation to STDs within the MSM population; two nursing researchers with expertise in the field of HIV and AIDS, as well as delivery and development of Web-based interventions; one computer and software research engineer; three sexual health community-based clinicians with access to the target population; individuals representing the target population; a Web design team to support the visual development of the Web-based intervention. | Patients  Single  The intervention composed of 3 sessions, each 60-minutes long, and participants have a total of 2 weeks to complete all 3 sessions. The first session will focus on planning condom use when having anal intercourse; session 2 will focus on negotiating the use of a condom with a partner; and the final session will focus on choosing not to have sexual intercourse without a condom. | NR |
| Muir  2017 [29]  UK  Eating disorders  Multiple (quantitative survey, randomized trial) | Needs assessment  The authors reviewed both their own qualitative research and other published work relating to patients’ experiences of having an eating disorder. Telephone-based service provision surveys were also used to understand the impact of nonattendance at specialist eating disorder services.  Program objectives  To increase attendance at an eating disorder service.  Theoretical framework  Motivational Interviewing; Self-Determination Theory (SDT); and Person-Based Approach.  Designing the Program  The authors adapted an already existing booklet designed by authors that aimed to help people think about the recovery process.  The program was specifically designed to be professional-looking, gender- and age-neutral.  Initial prototype of the program has already been evaluated through interviews with 12 participants with eating disorders to understand their beliefs about the relevance, acceptability, and usability of the program. The feedback was used to make modifications to the program.  Implementation planning  Two team members demonstrated the program to staff at a local eating disorder service and invited feedback about implementation.  Evaluation planning  A randomized controlled trial is also currently underway to evaluate the impact of the program on improving attendance at an eating disorder service. | People with eating disorders, researchers and clinicians designed the intervention. | Patients  Single  MotivATE: Four 15-minute Web-based modules – (1) address expectations about the assessment appointment; (2) introduce the idea of change; (3) help people to recognize problematic behaviors (precontemplation) and address ambivalence; and (4) improve confidence to attend eating disorder services. | NR |
| Puijk-Hekman  2017 [30]  The Netherlands  Cardiovascular disease (CVD)  Multiple (randomized trial, mixed methods research) | Needs assessment  Needs assessment followed the Predisposing, Reinforcing, and Enabling Constructs in Educational/Environmental Diagnosis and Evaluation (PRECEDE) model.  A literature search of perceived problems related to self-management of CVD. Three meetings of expert groups to prioritize the problems and identify the most important and modifiable underlying behaviours requires to self-manage CVD.  Program objectives  (1) Lifestyle (nutrition, physical activity, smoking and use of alcohol); (2) setting boundaries; (3) medication adherence; (4) emotions (fear); and (5) interaction with the health professional.  Theoretical framework  Behaviour Change Technique (BCT).  Designing the Program  All expert groups were elicited about their feedback on program design and implementation.  Implementation planning  All expert groups were elicited about their feedback on program design and implementation.  Evaluation planning  A mixed-methods study design will be conducted with an early randomized controlled trial and a process evaluation to (1) evaluate the potential effectiveness and effect size of Vascular View; (2) identify the outcome measures most likely to capture potential patient benefit; and (3) evaluate continued participation or withdrawal from Vascular View. Between 400 and 600 potential participants diagnosed with CVD will be recruited. | Two expert groups: (1) six patients, and (2) six health professionals (physicians, nurses, psychologists, dietician, and a physical therapist).  Expert groups met three times to ensure that the program is tailored to the perceived problems and the needs of patients and health care professionals. | Patients  Multifaceted  Vascular View program: comprehensive, multi-component, tailored, web-based, self-management support program. The program starts with a welcome for every patient, in which the different courses (Coping with CVD, Setting Boundaries in Daily Life, Lifestyle, Healthy Nutrition, Being Physically Active in a Healthy Way, and Interaction with Health Professionals) are explained. A patient may randomly visit and complete any course. In the welcome section, patients fill in a short questionnaire, which guides their choice of preferred courses.  Patients receive a written instruction manual for Vascular view and diaries are made available before receiving the codes to login. Digital flyer is sent to patients that promotes the benefits of using the program and contains contact information for relevant persons. | NR |
| Sakakibara  2017 [31]  Canada  Stroke  Multiple (qualitative NR, randomized trial) | Needs assessment  1) Establishing and working with a planning group to provide input throughout intervention development; and 2) conducting a detailed literature search to assess the issues associated with secondary prevention efforts and conceptualize a framework for an intervention to address the needs derived from theory, empirical evidence, and practical knowledge.  Program objectives  To develop a comprehensive and patient-centered intervention that would allow participants to focus on any behavioral risk factor (i.e., physical activity, diet and nutrition, stress management, smoking, and alcohol consumption).  Theoretical framework  Social Cognitive Theory; and Control Theory.  Designing the Program  Four strategies were in place. Authors conceptualized the 1) dose (seven coaching sessions over a 6-month period); 2) delivery (via Telehealth); and 3) organization of the intervention (5A organizational construct for clinical counseling). This was supplementing with obtaining 4) stakeholder feedback (i.e., end users and decision-makers from organizations that could implement the intervention).  Implementation planning  Initial implementation included the development of training materials for coaches (information on stroke, epidemiology, stroke risk factors, and the importance of lifestyle behaviour modification to improve secondary prevention, components of Stroke Coach, delivery of each of the telephone sessions, and review the coaching manual and practice) and methods to assess program fidelity (coaches record sessions for intervention developers to listen and critique).  Evaluation planning  The authors developed a research protocol detailing the evaluation using a multi-site single-blind randomized controlled trial study design. This will be coupled with a qualitative study. | The planning group (n = 8) comprised of health care professionals, including a neurologist, psychologist, physiatrist, dietician, and physical therapist, and researchers with expertise in neurosciences, stroke and cardiac prevention, human nutrition, behaviour change theories, self-management, and research and evaluation methodologies. Three members were leaders in their health authorities and could implement the program in their setting. | Patients  Multifaceted  Stroke Coach consists of three practical strategies: (1) lifestyle coaching, (2) self-management manual, and (3) self-monitoring kit. | NR |
| Shegog  2017 [32]  USA  Epilepsy  Randomized trial | Needs assessment  Established a planning group; created a logic model; literature reviews; (from theory, empirical findings, and participant involvement) to inform a logic model of change.  Program objectives  (1) To influence patient self-management behaviour and (2) to influence the mediating patient-provider communication regarding self-management.  Theoretical framework  Social Cognitive Theory; two self-regulation models; the 5-A’s Behavioral Change Model; and Motivational Enhancement Therapy.  Designing the Program  Refinement of the program’s structure and organization, planning for program materials, drafting of messages and materials, and pretesting, refinement, and production of materials.  Implementation planning  Describing potential program implementers, stating the outcomes and program objectives for implementation, constructing matrices of change objectives for implementation, and designing implementation interventions.  Evaluation planning  Developing effect and process evaluation questions, developing indicators and measures of assessment, and specifying an evaluation design.  The planned evaluation design for MINDSET involves an randomized trial with a sample of patients randomly assigned to treatment (MINDSET and usual care) and comparison (usual care only) groups (n = 30 per group) at three clinic sites over three visits to evaluate its efficacy. Each clinical site would recruit 20 patients to participate. | Planning group of general neurologists (n = 5), patients (n = 12), and a Patient Provider Advisory group. The Advisory group reviewed the content, assessed functionality, flow, and “low and feel”, tested the usability, and reviewed evaluation plans. | Patients + clinicians  Multifaceted  Management Information Decision Support Epilepsy Tool (MINDSET); decision-support system.  Originally mounted on an Archos 101 Android tablet platform (and subsequently on a Windows-based Dell platform), the use of MINDSET in the clinic comprises: (1) data entry by the patient; (2) data review by the patient and HCP; and (3) discussion by the patient and HCP of issues, goals, and strategies in conjunction with a tailored action plan. | NR |
| van Belle  2017 [33]  The Netherlands  Patient participation  Multiple (qualitative interviews, qualitative focus groups, program evaluation) | Needs assessment  Individual and focus group interviews with patients (n = 25) and nurses (n = 12); literature reviews.  Program objectives  Inviting the patient to use the Tell-us Card intervention.  Theoretical framework  Behaviour Change Theory.  Designing the Program  Program materials were tested with patients and nurses. The final layout was determined based on the original card and the comments of the nurses and patients. Additional tools to assist implementation were also developed during this stage.  Digital registration forms were developed in collaboration with an ICT assigned nurse, the IT department and the researchers (H & JC). An e-learning module was developed to inform and educate the nurses about patient participation, and the goal and use of the Tell-us Card intervention. The training ends with a short questionnaire to assess self-efficacy, as an estimation of efficacy predicts how nurses will actually deal with the intervention.  Implementation planning  Interviews with nurses enhanced the implementation strategies. Moreover, each ward had a group of nurses that guided implementation, stimulated the use of the intervention, and provided feedback.  Implementation strategies include an e-learning module, a core group of nurses as role models, visits to the ward for education, feedback and encouragement, informational letter for patients and nurses, and a kick-off meeting to encourage and education nurses.  Evaluation planning  The process analysis will follow a method in which evaluative data can be used to fine-tune the intervention (formative) as well as making a judgement about the extent to which the intervention was implemented (summative).  All Tell-us Cards would be collected by the researchers to examine the content and the number of patients reached with the intervention. | Patients and nurses were involved in the designing of the intervention and eliciting feedback on its layout. | Patients + clinicians  Single  Tell-us Card: a communication tool that aims to facilitate communication between nurses and patients. Patients write what is important for them on the card at the moment or in preparation for discharge from the hospital. Nurses read and acknowledge the patient preferences in the ensuing care. | NR |
| van Dongen  2017 [34]  The Netherlands  Nutrition and exercise  Multiple (quantitative survey, qualitative interviews, qualitative focus groups) | Needs assessment  A literature review, semi-structured interviews with researchers of original intervention (n = 2), semi-structured focus groups with professionals (n = 8), semi-structured interviews with participants of original intervention (n = 13), and semi-structured interviews with possible future participants (n = 9).  Program objectives  Participants initiate and maintain participation in the exercise and nutrition intervention.  Theoretical framework  Social Cognitive Theory.  Designing the Program  Four changes were made to the original intervention: (1) behaviour change goals for participants and health care providers were specified; (2) theoretical models were identified to ground the intervention; (3) a nutrition program with a dietician replaced the protein supplementation in the original intervention; and (4) a training program for implementing professionals was designed to ensure quality implementation.  A pilot study with one group (n = 25) using a pre- and post-test 12-week intervention trial.  Implementation planning  NR  Evaluation planning  NR | Implementing care organisations, the target group (frail older adults), and possible other stakeholders in the field of nutrition and/or exercise. | Patients + clinicians  Single  An intervention that consists of a combined resistance exercise and nutrition program for elderly participants and a training program for health care providers on recruitment and implementation. |  |
| van Dulmen  2017 [35]  The Netherlands  Cancer  Multiple (quantitative survey, qualitative interviews) | Needs assessment  Interviews with older patients (n = 10) to evaluate the intervention; literature review.  Program objectives  (1) To enhance patient participation during educational nursing encounters preceding chemotherapy; and (2) to improve older patients’ information recall.   Theoretical framework  Self-Management Education Theory.  Designing the Program  Usability tests were performed with older patients with cancer (n = 5) and older adults without cancer (n = 3).  Implementation planning  After a kick-off meeting at the research institute, two meetings per hospital were held to discuss the content and logistics of the study and its implementation, as well as to create support for the intervention.  Evaluation planning  A cross-sectional design will be used to evaluate the perceived usefulness and usability of PatientVOICE via an online questionnaire among older patients with cancer (65 years or older) who are receiving chemotherapy or have received chemotherapy in the preceding 5 years. | A coordinator was assigned at each hospital that became part of the project team and facilitated the contact with the 10 nurses that were interviewed.  Health care providers (e.g., nurses, assistants, team leads) of four hospitals were involved in the development of the intervention. | Patients  Multifaceted  Pre-chemotherapy online communication tool called PatientVOICE includes the following components: pre-counseling preparatory information, question prompt sheets, video modeling, and consultation audio recordings.  PatientVOICE contains a section with information on the nursing encounter preceding chemotherapy, including the question prompt sheet and video fragments, a section with information about chemotherapy, and a section that consists of a secured personal page for patients that includes the question prompt sheets and audio facility. | NR |
| Beentjes  2016 [36]  The Netherlands  Mental illness  Multiple (qualitative interviews, randomized trial) | Needs assessment  Literature review on health problems of consumers with severe mental illness; Google search for Dutch internet forums for people with mental illness; and content analysis of forum discussion; development group discussion; and intervention resources and literature.  Program objectives  (1) Having connections with other important people; (2) achieving personal recovery goals; and (3) reducing relapse of psychiatric symptoms.  Theoretical framework  Theoretical models of behaviour change; PRECEDE Model; Stress Vulnerability Model; Transtheoretical Model.  Designing the Program  Examined literature on telemedicine and severe mental illness and gathered information on information and communication technology partners who could deliver the intervention.  Implementation planning  Examined literature on telemedicine and severe mental illness to identify implementation barriers; structured interviews with consumers with severe mental illness (n = 52) on use of computers and the Internet.  Evaluation planning  An exploratory cluster randomized controlled trial in which institutions were stratified by inpatient and outpatient clinic, control group hard copy textbook, intervention group hard copy textbook and web site. | A development group that consisted of six consumers with severe mental illness who previously completed the intervention, an informal caregiver, and two professionals who have worked with the intervention; one of the professional is a peer support specialist. | Patients  Single  Illness Management & Recovery (IMR) program: a standardized curriculum-based approach designed to provide consumers with severe mental illness information and skills necessary for managing their illness effective and working towards achieving personal recovery goals. This program consists of 11 modules with 56 chapters across 40 weekly sessions. | NR |
| Dalum  2016 [37]  Denmark  Smoking cessation  Multiple (qualitative interviews, randomized trial) | Needs assessment  Used practices from the U.S. Centers for Disease Control and Prevention and other major organizations were identified to obtain general recommendations on adult smoking  cessation interventions; a systematic literature search on digital smoking cessation interventions to identify key elements and recommendations for the intervention; and used the results of a survey on Internet use of the target group.  Program objectives  Performance objectives: decide to quit smoking, commit to the cessation attempt, prepare the cessation attempt, define smoking function, identify difficult situations, make a coping plan, plan new activities as smoke-free, set a quite date, quit smoking, use coping plan in real-life situations, evaluate and adjust strategies, maintaining quitting over time.  Theoretical framework  Self-Regulation Theory; Transtheoretical Model; Social Cognitive Theory; and Appreciative Inquiry.  Designing the Program  A plan was created by the planning group to translate knowledge and input from previous intervention mapping steps into an Internet- and text-based program. A smoking cessation group with smokers from target group was created. This group was followed and video-filmed throughout their quitting process. The program was drafted in plain language, with detailed descriptions of all elements and functions. A Web-programming company translated this program description into a specification plan. Functionality tests were performed with members of the planning group.  Implementation planning  The program was tested for three months before launch. Subsequent to launch, semi-structured interviews (n = 9) were held in February 2010 with users aged 23 to 60 years who were ex-smokers and had been enrolled in the Dit Digitale Stop Program (Your digital quitting program) for a minimum of 8 days.  Evaluation planning  Randomized controlled trial of the effectiveness of the program. The trial was conducted in a population-based sample of adult smokers in 2012-2014, and analysis of results is in progress. The control group received a self-help booklet. | Planning group comprises of interventionists and researchers at the Danish Cancer Society, which included individuals with expertise in web communication, managing a digital smoking cessation intervention for adolescents, generally communication and communication theory, smoking cessation counseling, theoretical understanding of the behavioural change processes, and systematic intervention development.  Steering committee consisting of representatives of the funders and staff responsible for implementing the program. | Patients  Multifaceted  Dit Digitale  Stop Program (Your digital quitting program). Each individual had their own web page with the following content: profile, my page, exercises, blog, videos, urgent assistance, my quit plan, and library. The program consisted of active follow-up and passive communication via the web page. | NR |
| Greaves  2016 [38]  UK  Heart failure  Multiple (qualitative focus groups, qualitative interviews, quantitative survey, feasibility study) | Needs assessment  Qualitative and quantitative literature reviews about self-care support needs of patients and caregivers; two *de novo* systematic reviews were undertaken by the project team (i.e., a meta-ethnographic synthesis of qualitative literature on the attitudes, beliefs and expectations of people with heart failure receiving cardiac rehabilitation; and a systematic review and meta-analysis of the efficacy and safety of cardiac rehabilitation in people with heart failure with preserved ejection fraction); consultation with experts in the field, including the project management group; reviewed the national and international clinical guidelines for heart failure recommended by the project management group; focus group interviews with two community-based heart failure support groups and attendees at a hospital-based rehabilitation class; face-to-face interviews with caregivers; discussion with the patient and public involvement group; and a needs assessment questionnaire was circulated to ten people with heart failure and 24 “experts in the field” (two behavioural scientists, 14 specialist nurses (heart failure, cardiac rehabilitation and primary care cardiac nurses), two cardiologists, two GPs, two exercise physiologists with cardiac rehabilitation experience, and two pharmacists).  Program objectives  Patients with Heart Failure:  (1) Engaging in exercise training to build (and maintain) cardiovascular fitness; (2) managing stress, breathlessness, and anxiety; (3) heart failure symptom monitoring (and associated help-seeking); (4) taking prescribed medications; and (5) understanding heart failure.  Caregivers:  (1) To facilitate improvement in quality of life for the person with heart failure by helping them to achieve the core priorities for change; and (2) to improve the quality of life for caregivers by acting to maintain their own health and well-being.  Theoretical framework  Leventhal’s Common Sense Model; Self-Determination Theory; and Control Theory.  Designing the Program  The output from the first three stages of the intervention process was used to generate intervention materials and a training course for facilitators. A training manual/syllabus for a 3-day training course for REACH-HF intervention facilitators was developed.  The patient and public involvement group commented in the program materials on their format and content.  A feasibility study was conducted to assess the feasibility and acceptability of the intervention, extract ideas to help refine the intervention, and assess the quality of intervention delivery. The REACH-HF intervention was delivered to patients (n = 23) and caregivers (n = 12) by seven trained facilitators at four sites (Cornwall, Abergavenny, Birmingham and York).  Implementation planning  Facilitators were trained using patient-centred counselling techniques on to tailor intervention components to the needs and priorities of each patient and caregiver.  Evaluation planning  NR | Patient and Public Involvement group consisting of six people with experiences of heart failure and three caregivers of people with heart failure. | Patients  Single  The Rehabilitation Enablement in Chronic Heart Failure (REACH-HF) intervention, a comprehensive self-care support programme.  The Heart Failure Manual - a written self-help resource for use by patients and their caregivers; The Progress Tracker - an interactive booklet designed to facilitate learning from experience/over time and the building of understanding about how self-care activities impact on symptoms, emotional well-being and quality of life, through practice, self-monitoring of progress and (facilitated) problem solving; the Family and Friends Resource - a manual for use by caregivers; and a training course for facilitators. | An additional file reports the results of the feasibility study: the manuals are well-accepted by patients, caregivers, and facilitators; patients and caregivers were highly satisfied with the intervention; there is a need to modify the manual content and format and the facilitator training; the intervention was indicated to be adequately delivered by all facilitators; two items need reinforcement in future interventions; excellent levels of outcome completion; patients and caregivers perceived low outcome burden; many patient and caregivers showed improvement; no safety issues; one of the tests was not population and failed to be effective. |
| Hall  2016 [39]  The Netherlands  Diabetes and Diabetic Retinopathy  Mixed Methods Research | Needs assessment  A mixed methods approach was used. Five psychometric measures, Diabetes Knowledge Questionnaire, Diabetes Health Beliefs, Self-Efficacy scale, Problem Areas in Diabetes Scale, and Hopkins Scale Checklist-25 and structured interviews (n = 26) relating to self-efficacy, addressing disclosure of living with diabetes and life-style changes were used to triangulate the quantitative findings.  Program objectives  NR  Theoretical framework  NR  Designing the Program  NR  Implementation planning  NR  Evaluation planning  NR | NR | NR  NR  Kilimanjaro Diabetic Program: details not reported. | NR |
| Jones  2016 [40]  Australia  Acquired Brain Injury (ABI)  Multiple (qualitative NR, quantitative NR) | Needs assessment  Literature review of physical activity after ABI, theories of behavior change used to change physical activity behavior, and the self-management of physical activity after ABI; a systematic review of the efficacy of current self-management programs; a literature review on the barriers to physical activity; and an online survey of a sample of Australian adults (n = NA) living in the community with an ABI was conducted.  Program objectives  Increase physical activity and reduce sedentary behavior.  Theoretical framework  Social Cognitive Theory; Transtheoretical Model; Health Beliefs Model; and the Theory of Planned Behaviour.  Designing the Program  The scope of the program was refined to providing six core lessons, released over eight weeks, with a defined sequence that was established through brainstorming and team planning sessions. The lessons were sequenced to provide the core content of the myMoves program, and were each accompanied by detailed case stories, providing the opportunity for modeling, and a worksheet to enhance skill acquisition.  Implementation planning  For the myMoves program, a website was designed and developed to provide both a source of information about the myMoves program and a point of contact with myMoves team members. This website provided an easy link between organizational adopters, potential participants, and the program implementers.  Evaluation planning  Both quantitative and qualitative methods were utilized. The results of this feasibility study will be used to inform further improvements to the myMoves program itself and the evaluation plan. | Potential participants and program adopters were involved in the program design and feedback. | Patients  Multifaceted  myMoves program: remote self-management program aimed at increasing physical activity; complex physical therapist intervention. | NR |
| Kerstenetzky  2016 [41]  USA  Transition of care setting  Multiple (qualitative interviews, document [EHR] analysis) | Needs assessment  Development of a logic model through a literature review on the topic of transitioning in health care settings; quantitative analyses (medication discrepancies, and discharge rates); and interviews with nurses/case coordinators (n = 69) and pharmacists (n = 50) regarding continuity of medication information.  Program objectives  NR  Theoretical framework  NR  Designing the Program  NR  Implementation planning  NR  Evaluation planning  NR | NR | Clinicians  NR  A guide to conducting targeted needs assessments (details NR). | NR |
| Norris  2016 [42]  South Africa  Pre-pregnancy  Multiple (qualitative not identified, Delphi process, randomized trial) | Needs assessment  (1) A stakeholder engagement and Delphi consensus study (n = 33); (2) semi-structured interviews with community leaders (n = 21), health care providers (n = 12), and young couples (n = 18); and (3) a literature review of clinical guidelines and systematic reviews on type 2 Diabetes and its underlying determinants.  Program objectives  Improve the health of young adult couples in Malaysia prior to conception.  Theoretical framework  Theory of Triadic Influence (TTI).  Designing the Program  Small working group developed a plan to implement the intervention. Presentations about the intervention were modified and then presented to community representatives, religious leaders, non-governmental organizations, and representatives of health services for comment and input.  Feasibility study of the trial was conducted with observation, focus groups, and semi-structured interviews.  Implementation planning  A wider campaign to promote the project and implementing Wifi hotspots in study clinics to enable participating couples to download the Jom Mama application.  Evaluation planning  A trial evaluation protocol was formulated that will enrolled Malaysian pre-pregnant women (n = 264) who are nulliparous not diagnosed with type 2 Diabetes and possess a smartphone with their spouses. | Small working group of authors developed the intervention plan to achieve behaviour outcomes. | Patients  Multifaceted  "Jom Mama" intervention; health system centred approach combining both community and electronic-health platform. | NR |
| Romeike  2016 [43]  The Netherlands  Nutrition and physical activity  Multiple (qualitative focus groups, randomized trial) | Needs assessment  Focus group interviews among the target group; review of the literature.  Program objectives  Promote a healthy diet and sufficient amount of physical activity among lower-educated Dutch, Turkish, and Moroccan adult women living in the Netherlands.  Theoretical framework  Theory of Planned Behaviour; and Self-Regulation Theories.  Designing the Program  Two versions of a computer tailoring intervention were developed: tailoring to sociocognitive variables (traditional tailoring intervention) and one that additionally includes tailoring to the ethnic identity to contextualize the intervention material (EI-tailoring intervention).  Logic algorithms were used in the online tool to produce personalized feedback for participants. The layout of the intervention material was designed by a graphic designer.  Two pretest phases. In the first, women were shown tentative drafts of the intervention material and asked to evaluate draft advice messages, pictures used in the brochures, and the layout of brochures. Women were interviewed about the likability, readability, attractiveness, and usability of the material. In the second phase, Dutch and Turkish women assessed the intervention delivery process by filling out a structured questionnaire eliciting their feedback.  Implementation planning  NR  Evaluation planning  The two versions of the intervention will be evaluated in a randomized controlled trial with three study roups: traditional tailoring, EI-tailoring and a control group receiving generic, nontailored information. The aim is to recruit 1000 participants (500 Dutch, 250 Turkish, and 250 Moroccan). A process evaluation will be conducted at three-month follow-up, assessing the self-reported use, relevance and appreciation of the intervention. | NR | Patients  Multifaceted  Two computer-tailored nutrition and physical activity interventions: traditional tailoring and EI-tailoring. In traditional tailoring, participants are provided feedback to their behaviour delivered in three printer brochures.  The first two brochures contain advice about two of the following behaviors: 1) fruit consumption, 2) vegetable consumption, 3 snack consumption, 4) PA. The recipient receives feedback on only two of the four behaviors, as addressing too many behaviors at a time may lead to ego depletion and because self-regulation might be a limited source. The third brochure is a follow-up brochure containing follow-up feedback on the behaviors addressed in the first two brochures. | NR |
| Shakibazadeh  2016 [44]  Iran  Diabetes  Multiple (quantitative survey, qualitative NR, randomized trial) | Needs assessment  Previously published qualitative studies; and a an unpublished survey of different stakeholder groups.  Program objectives  (1) Healthy eating, (2) being active, (3) monitoring blood glucose, (iv) taking medication, (4) problem solving, (5) reducing risks and (6) healthy coping.  Theoretical framework  Adult Learning Theory; Health Belief Model; and Social Cognitive Theory.  Designing the Program  A protocol for use by the program team to manage sessions was produced. Moreover, a package of patient materials was produced including forms for assessment, goal-setting, blood glucose monitoring, and ‘pros and cons’ exercise, information brochures and quick reference pamphlets on diabetes care, and patient’s HbA1c test results letter.  Implementation planning  NR  Evaluation planning  A randomized trial with program participants (n = 280) to assess diabetes knowledge, self-care activities, psychological and clinical outcomes. | Health care professionals involved in the care of diabetes patients. | Patients  Single  Persian Diabetes Self-Management Education (PDSME) program: standard self-management education protocol. This program comprises of 8, 2.5-hour educational workshops followed by two “booster” sessions. | Key improvement was a reduction in HbA1c (about 1%) at 18-21 months follow-up. Mean knowledge scores did not change in control group but increased from 0.24 to 0.39 (p < 0.001) for patients treated with oral antidiabetic agents in the intervention group. The knowledge of insulin users did not change. No difference in PDSME patients and those with usual care in the number of days each week adhering to general diet. Significant difference in health beliefs score between two groups; the intervention group’s positive attitude towards diabetes increased and negative attitudes decreased more in PDSME patients. |
| Smith  2016 [45]  USA  Breast cancer  Multiple (qualitative interviews, qualitative focus groups) | Needs assessment  A literature review; secondary data analysis of the National Health Interview Survey Cancer Control Supplement; lifestyle assessment survey (n = 240); and focus groups (n = 42).  Program objectives  Change objectives include: 1) be as lean as possible without becoming underweight; 2) be physically active for at least 30 minutes every day; 3) avoid sugary drinks and limit consumption of energy-dense foods; 4) eat more of a variety of vegetables, fruits, whole grains, and legumes such as beans; 5) limit consumption of red meats and avoid processed meats; 6) if consumed at all, limit alcoholic drinks to two for men and one for women a day; and 7) limit consumption of salty foods and foods processed with salt (sodium) by substituting with herbs and spices high in phytochemicals (e.g., basil, turmeric, paprika, thyme, and dill).  Theoretical framework  Health Belief Model (HBM); and Theory of Planned Behaviour (TPB).  Designing the Program  Included participatory engagement. SISTAAH Talk members reviewed themes from focus group discussions during three telephone support group meetings with the principal investigator. They then engaged with researchers in videotaped and audiotaped experiential cooking and exercise sessions, which will serve as content for the app.  A feasibility trial with SISTAAH Talk coaches (n = 12) and research-to-practice demonstration will be conducted. The feasibility trial will be assessed through the Reach, Efficacy/Effectiveness, Adoption, Implementation, and Maintenance (RE-AIM) framework.  Implementation planning  NR  Evaluation planning  NR | Community Advisory Board (CAB), with representatives from community coalitions and breast cancer support groups in Miami, Chicago, Houston, Los Angeles, and Philadelphia. | Patients  Multifaceted  Mobile cancer prevention app: experiential educational sessions with SISTAAH Talk coaches. Cooking demonstrations followed cancer prevention guidelines relative to nutrition and dietary intake. Exercise sessions focused on guidelines specific to physical activity. | NR |
| Steffen  2016 [46]  USA  Health education  Randomized trial | Needs assessment  Literature review of prevalence and correlates of medication nonadherence in older adults, the role of family involvement in medication-related behaviours, and health education for intergenerational caregivers.  Program objectives  (1) Increase caregivers’ knowledge and use of effective medication management strategies; (2) decrease medication-related hassles perceived by women caring for an older relative; and (3) decrease rates of adverse medication-related events for older care recipients.  Theoretical framework  Social Cognitive Theory (SCT).  Designing the Program  Detailed course outlines of the relevant information were created first. Then, the authors deliberated to develop a creative approach through brainstorming sessions. They decided on delivering the intervention at a diner with a wise and seasoned waitress serving as the primary character. Through interactions with various customers, viewers would be able to see various health care challenges being overcome. The intervention scripts were then written based on the content and creative approach. The scripts for each module were reviewed by expert consultants on their relevance, use, and goals. The script was then beta tested with a small group of lay persons unaffiliated with the intervention. These persons assessed the scripts on its readability and clarity. Detailed storyboards were created based on the scripts for the Web-based intervention (most common situations and needs of intergenerational female caregivers). The intervention was made accessible through a WordPress site.  The overall appearance of our site is largely the same for participants in both the Narrative Vignette and Comparison Didactic conditions. After clicking on one of the primary content areas, participants are presented one of the submenus. Although participants in both groups have access to the didactic portable document format (PDF) informational sheets and talking head expert videos presented in the left column, only participants in the Narrative condition have access to the scripted vignettes. Although all links are visible throughout the entire intervention period, we sequentially enable them throughout the course of an individual participant’s 4-week trial. Participants are free to choose to view the information in any order and spend as much or little time on the site as they would like.  (1) Attractive and effective with families assisting older adults enrolled in home health care (a medically vulnerable population) and is (2) feasible to embed within routine home health services for older adults. The authors aimed to evaluate the intervention’s impact on family–clinician communication and reported caregiving self-efficacy and role overload.  The authors developed a preliminary evaluation with a community sample of adult women providing informal care to an older adult.  Implementation planning  NR  Evaluation planning  NR | Working group established from units on campus departments, a community advisory board, and an interdisciplinary panel of consultations to assist with content and dissemination.  We had a team of expert consultants including pharmacists and nurse practitioners, as well as social work, psychology, and public health professionals consult with us about the most common difficulties associated with health care and medication management in older adults. This group of people supported the creation of detailed course outlines. | Patients  Single  The Sandwich Generation Diner: a tool for intergenerational caregivers of older adults with physical and cognitive impairments: features four modules that address specific health care concerns. | NR |
| Gray  2015 [47]  USA  Inflammatory bowel disease  Multiple (quantitative survey, qualitative interviews, qualitative focus groups) | Needs assessment  Two focus groups per each separate class of participants (patients n =15, parents n = 16, medical personnel n = 13) for a total of six focus groups; before the interview all participants completed a demographic questionnaire designed for the study to obtain basic background information.  Program objectives  NR  Theoretical framework  NR  Designing the Program  NR  Implementation planning  NR  Evaluation planning  NR | NR | NR | NR |
| Highfield  2015 [48]  USA  Mammography  Observational study | Needs assessment  (1) Assess organizational capacity; (2) conduct a needs assessment and develop a logic model of the problem; (3) develop a logic model of change; and (4) write program goals for expected outcomes for implementing the intervention at the new site. Details of the needs assessment published elsewhere and include literature review, interviews, focus groups, and survey.  Program objectives  Find, adapt, implement, and evaluate an evidence-based public health intervention to improve mammography adherence for African American women in a new practice setting.  Theoretical framework  Social Cognitive Theory.  Designing the Program  The team performed a second search of databases of peer-reviewed studies to find reports of the original evaluations conducted on the EBIs they had located; using the peer-reviewed articles that described the program and their evaluations, the planning group assessed the basic fit.  (1) Judge how well the candidate intervention fits the desired behavioural and environmental conditions from the community’s logic model of change; (2) judge whether the determinants of behaviour and environmental conditions and the change methods used to influence them in the original intervention are adequate in the new setting; (3) judge how well original delivery, design features, and cultural elements fit the new setting and population; (4) judge the fit of implementation strategies to the new setting; and consider which intervention elements are essential and decide how to retain them; (5) prepare design documents for the adaptations and drafting changes; (6) pretest the adapted materials; and (7) produce final adapted materials.  The planning team noted the planned strategies, described the program materials and activities in which change should be made and then wrote or edited messages that supported the change; pre-tested the scripts with local African American women (14 pre-testers worked in pairs to role play the scripts with one woman as counsellor and the other, as patient); the pre-testers noted changes in the scripts to make them as relevant as possible to local women; the pre-testers then thoroughly debriefed the role plays with the entire project teams and recommended that the calls the made by a culturally congruent counselor; the team produced the revised manual for barriers and foundational conversational scripts in the hard copy form.  Implementation planning  (1) Identify implementers, implementation behaviours, and outcomes; (2) develop implementation and maintenance scope, sequence, and instructions; (3) plan activities to motivate and train implementers; and (4) plan logistics including budget, staffing, and materials.  Planners compare the implementation protocol of the intervention to implementation considerations and constraints for the new site to create a revised protocol.  The team identified implementers: patient navigators, community health workers, clinical partner managers; the mammography team developed training to encourage self-efficacy, outcome expectations, and skills of the implementers.  Evaluation planning  (1) Write evaluation questions; (2) choose indicators and measures; (3) choose evaluation design; and (4) plan data collection, analysis, and reporting.  The authors employed a type 1 hybrid design to test the intervention’s effectiveness and father information on the implementation. A quasi-experimental, sequential recruitment design was used in which the authors assigned contacted women to usual care or adapted intervention in groups of 50 patients. The authors calculated descriptive statistics and then conducted logistic regression analysis to report attendance in the intervention group as compared to the comparison group while controlling for potential confounders. | The Charities organization initiated planning for the project; the planning group (representatives of the lead agency research arm, breast cancer provider organizations, the local Breast Health Collaborative, and the local school of public health) conducted a needs assessment; community data collection with local women; the planning group converted the logic model of the problem to a logic model of change to create a foundation for comparing evidence-based public health interventions to the needs in the community. | Patients  Single  The adapted evidence-based public health intervention consisted of one call per woman by a patient navigator or community health care worker aiming to increase mammography among African American women. | NR |
| Cabassa  2014 [49]  USA  Mental Illness  Qualitative (participatory action research) | Needs assessment  Mixed-methods needs assessment at study site to understand Hispanic clients’ physical health problems and experiences with primary care services. Data collection included structured interviews, medical chart abstractions, and five focus groups (n = 24).  CAB had 6 meetings; the research team developed a logic model visually illustrating the CAB's ideas, which was then presented to the CAB; the CAB provided input to the design of the needs assessment conducted by the research team.  Program objectives  CAB had 8 meetings where they conducted critical reviews of each of the intervention's core components, activities, and methods in order to identify areas for adaptation; the research team developed and presented matrices of change objectives; the CAB reviewed the intervention's logic model to identify and discuss areas for adoption.  Theoretical framework  NR  Designing the Program  (1) Review the objectives, methods, materials, and theoretical foundations of the intervention; (2) identify specific adaptation to intervention content or delivery; (3) incorporate adaptations into the intervention manual and materials; and (4) finalize adapted intervention.  CAB had eight meetings that included review and adjustments of intervention manual and materials.  Implementation planning  NR  Evaluation planning  NR | Community Advisory Board (CAB) comprised of a primary care physician, the clinic's director (a psychiatrist), several mental health providers (a social worker, an RN, and a peer specialist), and members of the research team (principal investigator and two research assistants); CAB met approximately on a monthly basis at the local clinic for a total of 26 meetings; on setting the stage step: CAB had 4 meetings that included icebreaker activities, mission statement development, and group discussions. | Clinicians  Multifaceted  PCARE adaptation: an intervention manual that includes: the use of bilingual health-care managers to deliver the intervention; a section discussing the importance and rationale for incorporating cultural norms into health-care manager interpersonal skills and interactions with clients; examples of behaviors that demonstrate and reflect cultural norms; DSM-5 Cultural Formulation Interview adapted for health problems; clients' health education materials; personal health record (PHR) as a client education and activity tool; client activation checklist to the PHR; problem solving module; modification of preventive care tracking tool by adding basic medical information; a coordination plan to assist health-care managers in tackling the local complexities of coordinating care with multiple doctors and community clinics; a training curriculum for social workers. | NR |
| Geidl  2014 [50]  Germany  Chronic diseases  NR | Needs assessment  Literature review.  Program objectives  Promoting adherence to exercise therapy and regular physical activity.  Theoretical framework  Integrative Model of Motivation and Volition for the Initiation of Regular Health-Enhancing Physical Activity; The Health Action Process Approach; and The Motivation-Volition (MOVO)-Process Model.  Designing the Program  NR  Implementation planning  NR  Evaluation planning  NR | Professors at a German institution gave ideas and supported the conceptual development of BET; another professor proofread the manuscript; German Pension Insurance funded exercise therapy projects that considerably contributed to the development of this paper. | Patients  Single  Behavioural Exercise Therapy (BET): complex, physical activity-based intervention that applies and integrates biomedical approaches to optimize a person’s capacity, approaches that build on and strengthen personal resources and develop a person’s performance in the interaction with the environment. | NR |
| Hesselink  2014 [51]  Netherlands  Hospital discharge  Multiple (qualitative interviews, focus groups, and artefact analysis) | Needs assessment  Literature search on the frequency and consequences of ineffective hospital discharge problems; a large qualitative study published elsewhere which conducted individual interviews, (n = 321), focus groups (n = 26), process maps (n = 5), artefact analysis (n = 5), and Ishikawa/fishbone diagrams (n = 5), where the stakeholders were discharged patients and their health care providers.  Program objectives  To evaluate the discharge process through hospital readmission rates and adverse event rates after the hospital discharge.  The performance objectives and matrices were discussed in a multidisciplinary study panel (n = 5) that included experts in health, social, and organizational sciences; member of the European HANDOVER Research Collaborative (n = 15) prioritised the large number of determinants of importance on a survey.  Theoretical framework  Social Cognitive Theory; Transtheoretical Model, Intergroup Contact Theory; Rational Decision-Making theories; Goal-Directed Behavior; and Theories of Integrated Care.  Designing the Program  Literature review of a list of evidence-based program strategies to achieve the objectives. No specification on which strategies were adopted for this particular program.  Implementation planning  A total of 220 international researchers, policy-makers, and regulators in the field of quality and safety in health care, health care providers, and patient representatives were consulted about their experiences with successful strategies or promising ideas during three expert meetings.  Evaluation planning  NR | NR | Patients + clinicians  Multifaceted  The intervention includes discharge templates, medication reconciliation, a liaison nurse or pharmacist, regular site visits and teaching-back. | NR |
| De Brito-Ashurst  2013 [53]  UK  Chronic kidney  Disease  Qualitative focus groups | Needs assessment  Literature review on the problem, and the population’s health and dietary behaviours; and qualitative focus groups to identify the barriers to and facilitators of dietary salt behaviour change and attitudes towards health, disease, and diet in this population. Qualitative findings are published in another manuscript.  Program objectives  Reduce dietary salt intake in Bangladeshi patients with chronic kidney disease and hypertension, with a view to reducing their blood pressure and slowing kidney disease progression.  Theoretical framework  PRECEDE Model; and Health Education Model.  Designing the Program  Local community centers, local community dietitians and Bengali key workers were contacted seeking local nutrition programmes, and especially any developed for the Bangladeshi population and their dietary behavior; a meeting with local Bengali key workers and community dietitians helped develop the program framework; a pilot Community Kitchens UK session was conducted with Bengali renal disease patients (n = 6), a trained Bengali facilitator, and the researcher whereby the format, recipes, and evaluation were discussed.  Construction of the program involved finding a location for cooking sessions and developing, translating, piloting, and producing low-salt dietary educational material.  Implementation planning  NR  Evaluation planning  NR | Project group comprising of researchers, interpreters, Bengali key workers and experts working in the field of Bengali dietary education. Brainstorming sessions were used to identify and plan nutrition educational programs most likely to influence behavioural change. | Patients  Multifaceted  The Community Kitchens UK program involved cooking sessions (at community centers) and translated low-salt dietary material; trained Bengali volunteers facilitate the sessions and decide on recipes and buy ingredients. The program consisted of tailored communication and reinforcing factors. | NR |
| Laisaar  2013 [54]  Estonia  HIV  Multiple (qualitative interviews, qualitative focus groups, randomized trial) | Needs assessment  Literature review; focus group sessions with individuals undergoing anti-retroviral therapy (n = 14) to identify barriers to ART adherence; interviews with medical service providers (n = 4).  Program objectives  To facilitate integration of anti-retroviral therapy into the context and demands of daily life; optimal anti-retroviral therapy adherence support for HIV infected persons initiating or already on anti-retroviral therapy, attending a medical institution.  Theoretical framework  Information-Motivation-Behavioral Skills (IMB) Theory; and Next-Step Counseling (NSC) to anti-retroviral therapy adherence.  Designing the Program  NR  Implementation planning  The intervention nurse and the interviewer received two half-day training sessions in July 2010 and 3 two-hour boosters over the next three months; the project manager supported the implementation through regular discussions with the study-nurses at least once a month.  Study material was in English, Estonian, and Russian and staff was trained in these languages. A study manual on NSC and Neutral Assessment approaches, a one-page NSC counseling record form, and a standard questionnaire for patient interviews were employed to standardize and monitor the implementation.  Evaluation planning  Intervention being evaluated in a randomized controlled trial with 150 patients attending the infectious diseases department at Ida-Virus Central Hospital. This study aims to evaluate anti-retroviral therapy adherence and the impact of the intervention compared to regular counseling. | Research team included: two public health researchers, experienced in HIV/IDU, medical doctors, researcher/instructor in health behavior change, experienced in anti-retroviral therapy adherence, a psychologist, two nurse counselors supporting the researchers in adapting the intervention to local conditions. | Patients  Multifaceted  Situated Optimal Adherence Intervention Estonia (sOAI Estonia): the intervention consists of three one-on-one sessions approximately three months apart with a trained study nurse; session 1 has three components: (1) an explanation of the study and the role of the nurse as a collaborator with the participant, (2) going through the educational flipchart and eliciting reactions to the content, and (3) a next-step counselling discussion; sessions 2 and 3 consist of a brief check on any questions arising from the first session, and implementation of the next-step counselling discussion. | NR |
| Munir  2013 [55]  UK  Cancer  Multiple (quantitative surveys, qualitative interviews, qualitative focus groups, Delphi process) | Needs assessment  Literature review to identify the employment and work-related issues in adult cancer patients and the existence of any work-related guidance tool; discussions with Macmillan Cancer Support, a key stakeholder involved in return-to-work (RTW) research and supporting employees with cancer to return to work; two focus groups with individuals from the Macmillan support group who had or were recovering from cancer. (n = 14) were used to explore additional gaps in the literature and inform the content and structure of the tool.  Program objectives  Enables patients with cancer to take the lead in stimulating discussion with a range of healthcare professionals, employers, employment agencies and support services.  Theoretical framework  Empowerment Theory.  Designing the Program  Literature review to identify existing empowerment tools and evaluate their content; using this information, the first draft of the tool was developed consisting of 43 questions divided into four categories (initial work issues, preparing to return to work, returning to work, and not returning to work) and three themes (health, finance, and work); two-round Delphi consensus process with various experts (n = 172). In the first round, each participant rated each question to the degree they thought that the question posed would help the target group make decisions related to work and well-being and to whom the question should be asked. In the second round, 139 participants, responded to the questions that did not reach consensus in the first round. A feasibility study was then carried out with an independent group of participants (n = 38) to assess whether the tool met the change objectives and potential strategies.  The guidance tool has also been evaluated by an independent research team at the University College of London.  Implementation planning  Currently being led by Macmillan Cancer support; an implementation plan and training for healthcare professionals, support groups, and employers who could direct those with/recovering from cancer to the tool; Macmillan printer 10,000 copies of the tool and distributed them in their network. Macmillan raised awareness of the tool and mobilised human resources personnel, line managers and employers for training on the usage of the tool.  Evaluation planning  For this study, the effectiveness of the tool will be evaluated using a randomized controlled trial. Individuals diagnosed with cancer and are employed at the time of diagnosis will be recruited (n = NR). A process evaluation will also be conducted to assess satisfaction and utility of the tool. | A steering group consisting of members of the National Cancer Survivorship initiative and Macmillan Cancer Support, and experts provided guidance regarding the scope and content of the tool. | Patients  Single  Self-guidance tool for patients with cancer to return to work. This tool contains guiding questions in different categories which represent the stages of the cancer journey in relation to work. | NR |
| Theunissen  2013 [56]  Netherlands  Chlamydia screening  Qualitative interviews | Needs assessment  Literature review of lessons learned from existing screening programs, and chlamydia prevalence in different populations; semi-structured interviews with chlamydia positive individuals (n = 21); they were interviewed about partner notification to find out the determinants to motivate peers for the Ct test.  Program objectives  To reduce the patient’s and population’s chlamydia burden and improving the timing of care.  Theoretical framework  Precaution Adoption Process Model.  Designing the Program  The selected applications were incorporated into two websites: (1) a smart and highly interactive website for young people to communicate and motivate those at high risk in their networks, and (2) another website for sexual health care professionals to start recruitment-chains and oversee the process of home-test requests.  The intervention was pretested and refined with close involvement of high risk young people and sexual health care nurses.  Implementation planning  A web-based Respondent Driven Sampling approach was used to recruit participants. Chain-referral sampling was also used. Moreover, to improve the chance of the target contact to decide for testing, messages and home-based test kits were used. Other methods to improve testing and motivating included tailoring, personalize risk, modelling, consciousness, raising, elaboration, mobilizing social support and self re-evaluation.  Evaluation planning  An evaluation plan was formulated with qualitative and quantitative measures. | Healthcare professionals (sexual healthcare nurses and medical doctors) and policy makers were involved from the start of the project by regular team meetings. | Patients  Single  The program consists of a chlamydia screening strategy using a web-based respondent driven sampling engine and an online interface specially written for young people to recruit and motivate peers; the website is named www.SafeFriend.nl; once they login into the website they will be offered information on sexual health and likely chlamydia risk; they will be prompted with a questionnaire and the answers are automatically assessed to decide to offer him or her a home-based chlamydia test. | NR |
| Byrd  2012 [57]  USA  Cervical cancer  Screening  Multiple (quantitative survey, participatory action research) | Needs assessment  Literature review; 13 focus groups with Hispanic women from El Paso (n = 84); qualitative data from focus groups was used to develop a quantitative survey to provide additional information. a quantitative survey was pilot tested in women (n = 50) from the priority population; the survey was administered in 500 door-to-door surveys of Hispanic women.  Program objectives  To increase cervical cancer screening in US women of Mexican origin.  Theoretical framework  Health Belief Model; Theory of Reasoned Action; Transtheoretical Model; and Social Cognitive Theory.  Designing the Program  Other community members reviewed needs assessment data collection tools for clarity; Community Advisory Board members, investigators, and public health students generated matrices of program objectives. The authors reviewed IM matrices with the Community Advisory Board and chose appropriate theoretical methods based on their input.  CAB suggested components of the intervention, how it should be delivered, and whether materials were culturally appropriate; another advisory group comprised of lay health workers and clinic directors added and refined intervention materials based on pilot-testing which consisted of two-half training workshops with lay health workers, and three one-on-one pilot educational sessions with Hispanic women.  Implementation planning  An implementation guide was developed but no details were provided.  Evaluation planning  Evaluation through a randomized controlled trial but details are not reported. | Community Advisory Board (CAB) comprised of 10 community practitioners, community-based organizations, lay community health workers, Hispanic women met quarterly, and recruited other community members who were involved in various aspects of IM such as reviewing what had been found in the survey research, clarifying what the data might mean, deciding what intervention methods would be most appropriate, giving input on the development of materials, and recruiting women to assess materials before final development. | Clinicians  Multifaceted  AMIGAS intervention: educational video; flip chart for use by lay health workers to reinforce video messages; list of providers in the area and how to access services; props (speculum, cytobrush); Pap test record card; lay health worker training manual and implementation guide on how to use the intervention materials; message cards for lay health workers to counsel individual women; group games; low literacy posters and handouts; commitment to screening declaration sheet. | NR |
| Cherrington  2012 [58]  USA  Diabetes  Multiple (qualitative interviews, randomized trial) | Needs assessment  Literature review of existing diabetes programs involving peers or lay health workers; semi-structured interviews with program managers from diabetes peer advisor programs across the country about the barriers and facilitators to implementation; local, informal needs assessment conducted among members of existing cancer community coalitions.  Program objectives  To improve diabetes self-management among African American adults with diabetes in Alabama’s Black Belt; improve glycemic control, blood pressure, and lipid levels through a peer advisor intervention designed to improve self-care, proximal measures, including self-care behaviours.  Theoretical framework  Chronic Care Model.  Designing the Program  Four-week pilot of the training and the intervention, where two peer advisors were trained, and seven participants were recruited; peer advisors met their clients on enrollment day to conduct the needs assessment and then called their clients weekly, peer advisors also had weekly calls with investigators to report on progress and troubleshoot problems; after the pilot study investigators conducted two 1-hour group interviews, with one of the peer advisors and one with one of the clients, providing them with the opportunity to discussion intervention fidelity, reinforce skills taught during training, and offer support.  Based on the pilot study, a peer support network was established to enhance retention and morale, minimize burnout, and permit case reassignment if a peer drops out. Monthly support meetings were planned during the intervention period and peer advisors were paired with each other.  Implementation planning  Information from the pilot test was used to modify the peer training manual so that it can serve as a guide for training sessions and as an ongoing resource during the intervention period and afterwards.  Evaluation planning  Program is being tested in a randomized controlled trial with more than 60 peer advisors have completed training, 424 participants have been enrolled, and 200 have been matched to peer advisors. A process evaluation is being performed concurrently with the randomized controlled trial. | A discussion group with seven peer advisors from previous cancer awareness projects in the Black Belt were held to solicit feedback on the structure of the peer advisor program, recruitment and retention strategies, methods for intervention delivery, and perceived needs. | Patients + clinicians  Single  Diabetes Peer Support Intervention: 2-day training for peer-advisors; one-on-one in-person needs assessment conducted via telephone; maintenance phase of at least once monthly contacts for the remainder of the intervention period.  Three 30-minute interactive learning modules led by nurses or trained health educators; the modules include: 1) diabetes basics and the office visit, 2) healthy eating, and 3) exercise and stress management. | NR |
| Cornelio  2012 [59]  Brazil  Hypertension  Multiple (analysis of administrative data, quantitative survey) | Need assessment  Previously conducted studies document the needs assessment results of this topic using a survey; the project group discussed the intervention components; pre-tested selected content for acceptability in women (n = 10) with characteristics similar to the target population to ensure that the practical applications selected were appropriate.  Program objectives  To reduce salt intake among hypertensive women.  Theoretical framework  Transtheoretical Model; and Social Cognitive Theory.  Designing the Program  Simulation activities where women had the opportunity to measure spoon for 4g of salt; reinforcement phone calls for verbal persuasion; and role-play to identify barriers and formulate appropriate coping responses.  Brainstorming sessions conducted with experts and members of the target group to identify the intervention materials and to refine the sequence of programme interventions; a manual of intervention was produced to guide the health professionals; a prototype of the programme was pilot tested with representatives of the target group (n = 10); at the end of pretest the women were asked through open questions about the strengths and weaknesses of the meetings, and the quality of materials used for changing their dietary behaviours related to salt intake.  Implementation planning  Developed a plan for accomplish program adoption and implementation, created the materials and developed a plan for the recruitment of participants, and trained the facilitators to carry out the intervention and they were informed regarding its practical applications.  Evaluation planning  A pre-post test randomized controlled trial was performed to evaluate the effects of the intervention on both motivational (self-efficacy and habit) and behavioural (intention) determinants and salt consumption behaviour. | NR | Patients  Single  The program developed was called "SALdável" and its main theme was "use no more than 4g of salt per day to prepare your food"; the program consisted of two 60-90 min group sessions, offered to a maximum of 10 women; with two reinforcement phone call; the first group session was conducted by two nurses and the second one by a nurse and a nutritionist; a manual of intervention was used to guide the health professionals (nurses and nutritionists) who were the facilitators of the intervention. | NR |
| Gillison  2012 [60]  UK  Cardiovascular risk  Qualitative interviews | Need assessment  In-depth interviews with patients (n = 11) recently diagnosed with metabolic syndrome from a single primary care practice; literature review of previous qualitative work; literature reviews on best practice guidelines for supporting people to change their diet and/or level of physical activity; and stakeholder consultation with the steering group.  Program objectives  To promote changes in diet and physical activity for people with high cardiovascular risk.  Theoretical framework  Health Action Process Approach (HAPA).  Designing the Program  Copies of the participant and facilitator handbooks, and detailed program specification were obtained from the intervention’s developers.  Implementation planning  NR  Evaluation planning  NR | Steering group that met quarterly (the group composed of 2 GPs, 2 patient representatives, 2 health psychologists, an exercise psychologist, a statistician, a consultant endocrinologist, a Research Development support manager, the Local Director of Public Health, and the research officer); the steering group provided referral pathways, facilities for physical activity, and forthcoming investment; the steering group finalized the initial screening criteria for the intervention to be adopted. | Patients  Single  The program Waste the Waist was designed based on the Greater Green Triangle programme; it consists of nine 2-hour group sessions delivered by lifestyle coaches (6 sessions based on empowerment ideology to emphasize the client's responsibility for making decisions and self-regulation; 3 maintenance sessions to provide support for up to 9 months) | NR |
| Noordegraaf  2012 [61]  Netherlands  Gynecological surgery  Multiple (qualitative focus groups, randomized trial, program evaluation) | Needs assessment  Literature search for behavioural and environmental conditions of prolonged sick leave and delayed return to work; patients (n = 21) participated in meetings and three focus group (each with 7 patients).  Program objectives  To empower gynecological patients during the perioperative period to obtain timely return to work and prevent work disability.  Theoretical framework  PRECEDE Model; and Attitude-Social Influence-Self-Efficacy (ASE) Model.  Designing the Program  Brainstorm session of the researchers to determine translate the theory-based methods into practical strategies; the project group convened at several meetings to invent various appropriate tools for the eHealth intervention; a website producer specializing in eHealth interventions and a screenwriter were consulted at some of the meetings; an experienced gynecologist outside the project team was consulted to judge the medical content of one of the tools.  Pilot test to evaluate the website by patients (n = 21), physicians (n = 22), eHealth specialists (n = 3), and one representative of a patient organization; each person completed the evaluation form regarding the demo version for the eHealth intervention, scoring on eight areas: appearance, behaviour prescriptions, burdens of using the website, content, delivery, message, participation, and assessment and tailoring.  Intervention was developed by the website producer with MODX, a web application framework.  Implementation planning  An implementation plan to enable an extensive evaluation of the intervention was developed and an appropriate linkage system for future implementation was composed.  A linkage system was created by involving future users and implementers of the intervention from the start of the intervention development process.  The researchers identified facilitating factors and barriers regarding adoption and implementation of the eHealth intervention.  Presentations with background information about the development of the intervention, its contents, and how to use it will be given to the gynecologists during general teaching meetings at their hospitals.  Evaluation planning  Through a randomized controlled trial design, the project group approached gynecology practices (n = 7) about participation in the evaluation with approximately 212 patients. The project group formulated inclusion and exclusion criteria for patients to participate in the study and developed appropriate outcome measures to evaluate the intervention's effectiveness, adoption, usage, and implementation. A process evaluation will occur concurrently to assess compliance, effectiveness, usage, barriers, and suggestions for improvement. | A project group consisting of 1 research physician, 2 gynecologists, and 2 occupational physicians.  A website producer specializing in eHealth interventions, a screenwriter, and a gynecologist were consulted at some of the meetings between project group members.  A committee with representatives of the Dutch medical boards of gynecologists, occupational physicians, and family physicians, and a representative of an umbrella patient organization were involved during the development of all steps of the intervention and agreed to stay involved during the final implementation steps of this intervention. | Patients + clinicians  Multifaceted  eHealth website: http://www.ikherstel.nl; consists of two main sections: An Action List to assist in resumption of activities (composition of a work-reintegration plan, resumption of normal activities, and evaluation of complications) and a central home page (videos, recommendations, FAQs, glossary, and links to other websites).  A separate section dedicated to physicians including guidelines, casuistry, and background information. | NR |
| Scarinci  2012 [62]  USA  Cervical Cancer screening  Multiple (quantitative survey, qualitative focus groups, program evaluation) | Needs assessment  Literature review; focus groups with Latina immigrants (n = 13) to explore sociocultural factors associated with cervical cancer and screening; qualitative interviews with Latino and Latina immigrants to explore the sociocultural factors associated with sexual risk reduction; and developed a quantitative questionnaire validates the qualitative data from focus groups and interviews among Latina immigrants (n = 202).  Program objectives  An intervention that focuses on primary (sexual risk reduction) and secondary (Pap smear) prevention of cervical cancer among Latina immigrants.  Theoretical framework  PEN-3; and Health Belief Model.  Designing the Program  Involved lay health educators (LHEs) for intervention delivery. The intervention was piloted among Latina immigrants (n = 10).  Implementation planning  Once the intervention was developed, the authors elicited feedback from LHEs and lay health advisors (volunteers) who were already participating in another program. In terms of retention efforts, the authors implemented multiple strategies that include contact with participants at least once a month: phone calls, quarterly newsletters, and quarterly pot-luck dinners. Operation and training manuals and strategies were finalized once the intervention was finalized.  Evaluation planning  Evaluation consisted of process and outcome evaluations. Process evaluation or treatment fidelity was addressed at five levels based on the NIH Behavior Change Consortium recommendations (study design, staff training, delivery of treatment, receipt of treatment, and enactment of treatment skills). Outcome evaluation included assessments of all components of the proposed theoretical models as well as the proposed primary and secondary outcomes. | Involved lay health educators (LHEs) for intervention delivery. | Patients  Single  The intervention consisted of eight sessions (six group sessions and two individual sessions). There was a behavioral cue associated with each session. In the first session, participants receive a “friendship box” to keep all the information cards as well as a bracelet. In each session, a charm corresponding to the knowledge and skill learned was added to the bracelet. | NR |
| Suzuki  2012 [63]  USA  Clinical preventive and screening services (Mammography, Pap tests, and clinical weight screening)  Multiple (qualitative focus groups, randomized trial, program evaluation) | Needs assessment  Literature review; a town hall meeting on health care access barriers with approximately individuals with various disabilities (n = 40); focus groups of adults with disabilities (n = 15) about barriers to receive clinical prevention and screening services.  Program objectives  To promote regular use of clinical preventive services by women with physical disabilities.  Theoretical framework  Health Belief Model; and Social Cognitive Theory.  Designing the Program  Literature search was conducted to confirm theoretical and empirical evidence for each proposed strategy. A peer trainer strategy was incorporated to promote self-efficacy.  A package of materials was (an activity workbook, informational brochures, and a copy of the presentation given by the trainers) will be distributed to each workshop participant at the beginning of the workshop to supplement training activities. These materials contained verbal messages to accommodate individuals with low literacy skills.  Program details were shaped by feedback form members of the priority population. The authors conducted a pilot study with individuals with disabilities (n = 15) during which all participants read the Kiles curriculum program manual before attending the workshop; three women with mobility impairments who participated in the pilot formed an advisory group, which reviewed the accuracy and understandability of the materials, including literacy appropriateness; the trainers examined the practicality of procedures and activities in the workshop and reviewed surveys.  Two peer trainers selected for the program that were very qualified because they had experience working with the population, were well connected to the disability community and also were women with a mobility disability of similar age to study participants; with only 2 highly qualified trainers, two women attended all training workshops and all follow-up phone calls.  Implementation planning  The authors adapted an existing program to be more relevant to the priority community. The workshop was condensed and more accessible for people with low literacy skills. The curriculum was prepared independent of geographical location. Also described the creation of the trainer’s manual assisting future program implementers to replicate the program.  Evaluation planning  A randomized controlled trial with volunteers (n = 230) who were assigned to the PATHS workshop group or control group. A process evaluation was completed with the randomized controlled trial to assess the strategies used by participants, intervention quality, and participant satisfaction with study procedures; participants completed surveys immediately following and 6 months after the workshop; the trainers completed a log of each monthly telephone call, where they reported intervention strategies described by participants as a useful for moving toward their performance goals. | NR | Patients  Single  The PATHS (Promoting Access to Health Services) intervention is a 90-120 minute workshop with structured monthly follow up contact for 6 months after the workshop; the workshop covers the topics: education about common conditions, risks of these conditions for women with a mobility disability, screening benefits, procedures, and recommendations, overcoming barriers of screening, building skills for communicating with physicians, setting goals, and initiating change. | NR |
| Zwikker  2012 [64]  Netherlands  Rheumatoid arthritis  Multiple (quantitative survey, qualitative focus groups) | Needs assessment  Literature review about the determinants of medication non-adherence in rheumatoid arthritis; questionnaires to patients with rheumatoid arthritis (n = 228) to examine the prevalence and possible determinants of non-adherence; two focus groups were held with non-adherent patients (n = 14).  Program objectives  To improve medication adherence to disease-modifying anti-rheumatic drugs in non-adherent patients with rheumatoid arthritis.  Theoretical framework  Health Belief Model; Integrated Change Model; and Motivational Interviewing.  Designing the Program  The intervention protocol was discussed during Task Group meetings. The intervention was piloted twice with 12 non-adherent RA patients.; the pilot was attended by an expert panel (2 psychologists) and a patient panel (independent advisory board of 2 RA patients familiar with scientific research).  Implementation planning  Rheumatologists and pharmacists responsible for carrying out the intervention in clinical practice were part of the Task Group. A practical workbook for use during the intervention was written for rheumatologists and pharmacists. To check treatment integrity, a plan is in place to analyze the audio-taped intervention sessions.  Evaluation planning  A single-blind, randomized controlled trial with non-adherent patients with rheumatoid arthritis (n = 120). | The Task Group was comprised of pharmacists, researchers, psychologists, rheumatologists, experts on motivational interviewing, and patients with rheumatoid arthritis. | Patients  Multifaceted  Two, 1.5-hour motivational interview-guided sessions with 5 to 7 rheumatoid arthritis patients of each led by a pharmacist and including a rheumatologist involving group discussion and a workbook for participants; an individual homework assignment between the two sessions; and a follow-up phone call by the pharmacist eight weeks after the last group meeting. | NR |
| Hanbury  2011 [65]  UK  Suicide  Mixed methods (time series analysis, mediational analysis, qualitative interviews, program evaluation) | Needs assessment  Semi-structured interview with one health professional from each of the eight community mental health teams participating in the study to explore Theory of Planned Behavior (TPB) constructs in relation to guideline adherence; a TPB survey was developed and piloted with a small sample of health professionals (n = 50) who were asked to provide feedback on the relevance and wording of the questions; survey was fully implemented with community health workers (n = 50).  Program objectives  Raising adherence to a national suicide prevention guideline.  Theoretical framework  Theory of Planned Behavior (TPB).  Designing the Program  An educational session was chosen as the most feasible type of intervention due to resource constraints.  Implementation planning  To increase accessibility, the intervention was delivered separately to each of the teams at their team bases.  Evaluation planning  Three techniques were used in the evaluation: (1) time series analysis of routinely collected audit data summarizing health professionals’ monthly rates of adherence to the guideline before, during and after delivery of the intervention; (2) mediational analysis comparing before and after scores across each of TPB constructs using a survey; and (3) qualitative process evaluation interviews. | NR | Clinicians  Single  An educational session consisting of (1) presentation containing persuasive message, (2) facilitated group discussion, and (3) vignettes of different guideline adherence situations. | The intervention did not have a significant impact on adherence; only scores of perceived behavioural control dimension were found to be significantly higher and more positive after the intervention; and guideline is perceived to be mandatory by community nurses. |
| Looijmans-van Akker  2011 [66]  2009 [67]  Netherlands  Vaccination  Multiple (quantitative survey, qualitative interviews, qualitative focus groups, program evaluation) | Needs assessment  Individual in-depth interviews with nursing home physicians (n = 3); two focus group sessions (n = 9); literature review on the determinants of influenza vaccine uptake among healthcare workers; two questionnaire studies to specifically assess these determinants of vaccine uptake among healthcare workers in Dutch nursing homes. The first questionnaire study was conducted with the management of 335 nursing homes in the Netherlands. The second study was conducted with two healthcare workers.  Program objectives  To enable researchers and practitioners to develop intervention programs tailored to their setting.  Theoretical framework  Health Belief Model; and Behavioural Intention Model.  Designing the Program  All intervention materials were developed by the study group; the information leaflet and posters were developed in collaboration with the design department of the University Medical Center Utrecht and the information leaflet was pre-tested by three nursing assistants (for clearness, meaningfulness, and usefulness and if information is missing from the leaflet); the data management section assisted the development of the website; the video was recorded in a nursing home by a professional cameraman from the design department of the University Medical Center Utrecht, in the video, a nursing home physician, a nurse, and a patient shared their experiences on influenza and influenza vaccination.  Implementation planning  Representatives of the Dutch association of nursing home physicians (Verenso) and the association of nurses and nursing assistants (V&VN) were approached to judge the different elements of the program; support for the program was given by Verenso, V&VN, and two other relevant healthcare management associations (Sting and ActiZ); plenary information meetings were held by specialised nurses of the local municipal health centre guided by a standardised protocol that included FAQs. To support future implementation of the program without assistance from the study group a step-by-step script of the total program was developed.  Evaluation planning  Effect and process evaluations were conducted. Effect evaluation evaluated the effectiveness of the program on influenza vaccine uptake through a randomized controlled trial of nursing homes (n = 33) and healthcare workers (n = 6636). In the process evaluation, compliance with the program components was measured. | NR | Clinicians  Multifaceted  The intervention program consisted of 3 components: (1) includes an outreach visit during which homes were to receive a step-by-step script of the program, all required materials, and background information on influenza vaccinations of healthcare workers (the required materials included: announcements, a personal invitation letter, leaflets, posters, and the reference to the programs' website; (2) consisted of the meetings with a plenary presentation, discussions in smaller groups, and a video with role models (meetings organized by specialized nurses guided by a protocol); and (3) prescribed the appointment of a physician as a local program coordinator to organize and promote influenza vaccination. | NR |
| van Der Veen  2011 [68]  Netherlands  Hepatitis B screening  Multiple (qualitative focus groups, randomized trial, program evaluation) | Needs assessment  Literature review of the health problem, behaviours leading to infection, and behavioural and cultural determinants; focus group discussions with members of the target population on factors related to HBV-screening behavior; a survey among respondents (n = 355).  Program objectives  Citizens of Rotterdam, who are born in Turkey and aged 16 to 40 years, if necessary, take hepatitis B, within 6 months after the start of the program implementation.  Theoretical framework  Tailoring Method; Evidential, Linguistic and Constituent-Involving Strategies; and the Betancourt's Model of Culture and Behavior.  Designing the Program  Literature review of suitable methods and strategies for addressing the specific determinants and change objectives. The authors organized a brainstorm session with experts in the field of tailored Internet interventions, during which best practices were discussed. Telephone lines were made available for those not having any access to the Internet.  A design document was developed that described the size and characteristics of the priority population, the idea of cultural tailoring, the important elements of the intervention, the logistic route of recruitment and the testing procedure, and a time frame. Based on this document, a website was designed by a professional designer; the tailored skins and peer pictures were pre-tested with Turkish people (n = 10); a trainee of Turkish origin was involved so that wording and cultural appropriateness was taken into account; cognitive interviewing about program components with members of the population (n = 20) and community leaders (n = 10); another pretest of the Dutch version of the website with participants (n = 33); pretest of intervention with health promotion Internet-intervention experts (n = 15); the Turkish version of the intervention was 20 times pre-tested by Turkish people (n = 4).  Implementation planning  Mass media attention, informing general practitioners about the health program, and program, and asking for participation using posters and flyers in waiting rooms were used as strategies for agenda setting.  Developers worked closely together with the health care staff of the Municipal Public Health Service in order to determine the best logical test procedure; the linkage group (representatives of key organizations, experts in the field of public health, and key persons in the Turkish community) gave feedback on the progress and plan further adoption and implementation during the development phase; other individuals at the Municipal Public Health Service were informed about the project during public health information meetings and a workshop.  Evaluation planning  Evaluation plan includes effect and process evaluations. The effect evaluation, through a randomized controlled trial, determined whether the culturally-tailored intervention was more successful in promoting testing when compared with a standard information intervention and a behaviourally trailed intervention. Process evaluation was carried out to collect data on the use, appreciation, and exposure of intervention. | Representatives of 12 organizations in the Turkish community were involved in the programme development from the start onwards, participated in poster and flyer dissemination, and discussed the existence of the programme during their regular meetings. A trainee of Turkish origin was involved in the whole process of program development. Community members were part of the pretesting phase and contributed to the linkage group.  The Municipal Public Health Service was the organization responsible for the development and implementation of the program. | Patients  Multifaceted  Website containing an internet intervention: (1) introduction to the health problem and aim of the website; (2) tailored test advice; (3) information and advice regarding screening, including perceived barriers and motivators; (4) obtaining laboratory form; and (5) information on laboratory locations. | NR |
| van Rijssen  2011 [69]  Netherlands  Disability  Multiple (quantitative survey, qualitative focus groups, randomized trial, program evaluation) | Needs assessment  Identified the needs of the Dutch Institute of Employee Benefit Schemes by consulting policy-makers with the most expertise of physician-claimant communication (n = 4); reviewing reports and publications of the Dutch Institute and allied health organizations; the needs of the claimants were assessed through a survey (n = 56) asking for comments on communication during an assessment interview they attended; a focus group study among social insurance physicians (n = 22) was used to assess the needs of the physicians (published elsewhere).  Program objectives  To improve the communication skills of physicians during work disability assessment interviews with disability claimants.  Theoretical framework  Social Cognitive Theory; and Kolb’s Model of Learning Styles.  Designing the Program  The training course developed was discussed and evaluated first with additional experts (n = 3)in the development and/or provision of training programs for social insurance physicians, and successively in a group of social insurance physicians (n = 15); the authors presented the research results and concept versions of the training course in meetings and asked the physicians to comment on its content and to provide further suggestions for improvement; the comments and suggestions were discussed with experts (n = 2) to establish the final programme.  Implementation planning  The implementation plan was done in collaboration with the experts (n = 2) from the Dutch Institute's Educational Department; a plan was made to promote the training course to potential participants; the implementation was supported by a manual developed for the teachers of the training course.  Evaluation planning  To formulate an evaluation plan, a brainstorming session with all authors was took place; these plans were presented to researchers (n = 30) and social insurance physicians/researchers (n = 15) who commented on the plans and explained what their choice would be; after consulting the staff and policy makers of the Institute with regard to feasibility issues, the authors made the final decision on the evaluation plan; the questionnaires for the evaluation plan were pilot-tested by social insurance physicians (n = 4) to access comprehensibility and relevance, and by two researchers who were familiar with the intervention to access whether the contents of the training course and the questionnaires matched.  A randomized controlled trial is planned to assess skills and knowledge. A process evaluation will also be carried out to determine the most effective and valued aspects, to identify barriers and facilitators for implementation, and to further improve the training course. | The first stakeholder was the Dutch Institute of Employee Benefit Schemes, which is the institute that employs most of the physicians performing work disability Assessments for entitlement to benefits (further referred to as ‘the Institute’). The second group of stakeholders consisted of medical disability claimants (in this study, employees who had been sick-listed for almost two years, applying for a long-term work disability benefit).  The third group of stakeholders consisted of physicians who were specialised in performing work disability assessments (in this study, social insurance physicians). | Clinicians  Single  Professional Claimant Communication: a two-day post-graduate communication skills training course was developed for physicians: included that includes theoretical information provision, role-play, feedback and suggestions, use of checklists to facilitate learning process, integration of top 10 most important research findings in the training courses, and a binder with handouts to remind physicians about their learning. | NR |
| Detaille  2010 [70]  Netherlands  Chronic Diseases  Multiple (qualitative focus groups, randomized trial) | Needs assessment  Focus group interviews conducted with employees with rheumatoid arthritis, diabetes, and hearing loss (n = 69) to explore the prerequisites for employees with chronic diseases to function at work; the same question was explored through concept mapping with health professionals (occupational health physicians, occupational health and specialist nurses, family doctors and specialists; n = 54); literature review to determine the prognostic factors related to work disability in employees with chronic somatic disease.  Program objectives  To help employees with chronic somatic disease cope with work-related problems associated with their disease.  Theoretical framework  Theory of Planned Behavior; and Protection Motivation Theory; and Goal-Setting Theory.  Designing the Program  Two additional sessions were developed on what is needed to be able to work with a chronic disease and how to communicate with supervisors, colleagues, and occupational health professionals about problems encountered at work. The program was developed to confirm the boundary limits of the original intervention.  Implementation planning  The promotion material and plan for recruitment of participants for training were developed. Participants for the course were recruited through the departments of Human Resource Management from companies, general practitioners and occupational health services in the region of Arnhem and Nijmegen in the Netherlands; an information letter and leaflet of the course were sent to companies (n = 82), general practitioners (n = 88), and occupational health services (n = 10) in both municipalities; the course must be facilitated by two moderators; one of them is to be trained at the University of Stanford to be a master trainer of the intervention.  Evaluation planning  A randomized control trial (n = 104) with eight months follow-up and a qualitative evaluation using focus groups with participants from two training groups (n = 15); at least 35 patients in each group. | NR | Patients  Single  Eight sessions, each 2.5-hour long: six of these sessions were from original intervention and two were additionally developed for the target population.  A course handbook for participants; a manual translated in Dutch; a manual for the facilitators including step by step instructions on how to implement the intervention; a questionnaire provided to the participants after 8 months of conducting the intervention and also for the control group. | NR |
| Koekkoek  2010 [71]  Netherlands  Behavior of non-psychotic patients and their clinicians  Mixed-Methods | Needs assessment  Literature review on ‘difficult’ patients; a qualitative interview study among patients (published elsewhere); a survey among mental care clinicians (published elsewhere); and a Delphi-exercise among scientists, policy makers, and expert-professionals (published elsewhere).  Program objectives  Prevention and management of ineffective behaviours by long-term non-psychotic patients and their treating clinicians.  Theoretical framework  Network Episode Model.  Designing the Program  The authors visited three well-known national best practices for data on possible effective practice-based strategies not yet described in the literature. Consulted an expert group of clinicians, scientists, and policy makers over 2 years.  Implementation planning  The steering group developed the implementation plan. The intervention was presented to a director, research psychiatrist, and the psychiatrist of the team in which the intervention would be implemented. The intervention and its evaluation were presented to the team members who all agreed to participate. Ethical permission and the final approval obtained from the institution's chief director; the implementation team consisted of 6 psychiatric nurses and 2 psychiatrists; the team leader (one of the clinicians) and the team psychiatrist functioned as the link between the treatment team and the research team; the intervention was implemented through a 3 day-training program which was offered by the first author, and four specialists in the specific skills; the training program was followed up by the first author by biweekly supervision sessions and hands-on support by email, telephone or face-to-face contact.  Evaluation planning  A mixed-methods pilot study including quantitative outcomes, process measures (service use, treatment satisfaction, and quality of the therapeutic alliance), and qualitative interviews to assess the feasibility and usefulness of the intervention. | Steering group of scientists and managers in the psychiatric service the intervention was tested in. | Patients + clinicians  Single  Interpersonal Community Psychiatric Treatment (ICPT) is to be used in departments or programs for long-term ambulatory care. This intervention consists of a 45-minute session between the clinician and the patient that includes: in the first 5 minutes the clinician and the patient agree on the agenda (goals) for the session (motivational interviewing is used) ; 5 minutes are used to look back at the previous session, 25-30 minutes the goals set in the agenda are discussed and summarized; the last 5 minutes are used to look back at the session and clinician fills out a report form, while the patient a feedback form. | NR |
| Schmid  2010 [72]  USA  Stroke prevention  Multiple (quantitative survey, qualitative focus groups, qualitative interviews) | Needs assessment  Semi-structured interviews with healthcare providers (n = 44) to elicit their needs and barriers to systematic delivery of secondary stroke prevention and preferences and suggestions for program elements and implementation strategies; focus groups with key stakeholders (the veteran stroke survivors and their caregivers) to understand their barriers and preferences for secondary stroke prevention services (published elsewhere.  For interviews, a team of health care providers and researches first reviewed and critiqued research questions with four providers and made modifications based on their recommendations; four providers pilot tested the interview questions and suggested recommendations; four experienced research staff trained by investigator conducted interviews with 44 providers.  Program objectives  To tailor and implement the use of available tools for secondary stroke prevention into an existing healthcare system.  Theoretical framework  Chronic Care Model; and Theory of Planned Behavior.  Designing the Program  Interview findings from needs assessment were also used to discuss proposed strategies to assess the acceptability of the program and to gain provide suggestions for implementation.  A standard information packet that included handouts and pamphlets addressing the risk factor modification was developed. A stroke risk factor checklist poster was developed and placed in neurology workstations. A stroke risk factor ‘prescription pad’ was developed to identify and prescribe appropriate resources for each of the stroke risk factors and contact information at their local facility. Standard training and education regarding patient motivational interviewing and goal setting to foster behaviour change and support. This training included role playing and materials and handouts to disseminate to their patients and caregivers. There is also a monthly stroke support group with activities such as nutrition, stress, and management.  Clinicians provided positive feedback on the prescription pad and providers requested the pad to be transferred into an electronic order; stroke survivors reported about secondary stroke prevention.  Implementation planning  Tailored the intervention to local needs and interests. Recruited a clinical champion at each facility to help assist with the implementation of the program and sustain it after the end of study funding. The authors also tracked how the components were delivered and used by the veteran and provider, where they were used, and the delivery format. They also included a patient self-management checklist that assisted in documented the activities that patients engaged in to manage their stroke risk factors.  Evaluation planning  Medical record interviews with providers to determine whether there was lifestyle or medication management counseling, or specific stroke prevention goals in the rehabilitation notes. Quantitative surveys administered with patients to determine stroke-related outcomes such as quality of life and stroke severity. | NR | Patients + clinicians  Single  Handouts and pamphlets addressing the risk factor modification given to all patients with stroke or transient ischemic attack by a specified nurse prior to hospital discharge; stroke risk factor checklist poster; stroke factor 'prescription pad'; standard training and education regarding patient motivational interviewing and goal setting given to providers; local stroke support group; training for the rehabilitation therapists to incorporate a stroke risk factor management goal for every patient with stroke or transient ischemic attack; self-management training for veterans to learn goal-setting techniques to modify his stroke risk factors to reduce his risk for secondary strokes. | NR |
| Albada  2009 [73]  Netherlands  Genetic counseling  Randomized trial | Program objectives  A needs assessment among counselees who were first in family seeking cancer genetic counseling (n = 128; published elsewhere using a survey).  Program objectives  Enhance counselees’ realistic expectations and participation during genetic counselling.  Theoretical framework  Elaboration Likelihood Model (ELM).  Designing the Program  Information was gathered from patients and counselees from brochures, standard counselee letters and web texts. A list of potentially relevant tailoring variables was developed. The expert panel decided on relevant tailoring variables and appropriate adaptation of messages to each tailoring variable; E-Info Gene was tested by counselees (n = 13) in breast cancer genetic counselling. A question prompt sheet was used to empower patients to ask questions about specific subjects. The intervention was developed online to be self-paced and private for individuals.  The intervention was edited in according with guidelines on web information. The text was adjusted to the readability of 4^th^ grade high school. All separately written messages were integrated into one coherent website. A question prompt was added which comes up when E-Info Gene is closed. The web page was then designed.  Respondents (n = 13) described their experiences in accessibility and understandability of the intervention. Evaluation form to be completed by counselees. The web page was made more attractive, and there were improvements in the web design, jargon explanations, and clarification in the content.  Implementation planning  NR  Evaluation planning  Randomized controlled trial will be employed with 200 consecutive breast cancer counselees at the Department of Medical Genetics of the University Medical Centre Urecht. | The expert panel (3 former counselees in breast cancer, 2 clinical geneticists, a genetic nurse, a psychologist, and an interdisciplinary social scientist) met three times a week and conducted one feedback round through email; the draft of E-Info Gene was circulated within the expert group and alterations were suggested; the draft was modified by the first author before being re-circulated; the web texts were discussed with counselors during departmental meetings; the department of Medical Genetics approved the final website. | Patients  Multifaceted  E-Info Gene: a website that provides breast cancer genetic counseling with computer-tailored information and a question prompt prior to their first consultation. | NR |
| Bartholomew  2009 [74]  USA  Heart Attack  Quantitative surveys | Needs assessment  NR  Program objectives  To disseminate the results of the Anti-hypertensive and Lipid-Lowering Treatment to Prevent Heart Attack Trial (ALLHAT) trial and the Seventh Report of the Joint National Committee on Prevention, Detection, Evaluation, and Treatment of High Blood Pressure (JNC 7) guidelines beyond the standard publication and presentation of trial results.  Theoretical framework  Social Cognitive Theory.  Designing the Program  The main dissemination messages articulated the treatment goals and expectations for hypertensive patients and were based on ALLHAT results and JNC7 report. The dissemination messages were reviewed and endorsed by the NHBPEP to acquire broad-based consensus from among organizations that represent health care providers who treat hypertension.  The intervention included a direct approach that intended to influence physicians through academic detailing and their professional societies, and an indirect method that intended to influence physicians through their patients and drug formulary systems.  A pilot phase was conducted with investigator educators (n = 24) for six months to test the feasibility of the intervention.  Implementation planning  Regional coordinators with clinical trial experience interviewed prospective educators to discuss their typical professional networks and the potential to reach providers beyond these routine contacts. Other considerations during the interview were: ability of the investigator to recruit patients, success in retention and adherence, potential as persuasive behaviour change educators.  Investigator educators were compensated $400 per presentation to cover purchase of a light meal or snack for the session participants, travel to venue, and an honorarium.  Evaluation planning  A process evaluation was designed to assess the reach and dose of the intervention. A pre- and post- questionnaire was also administered with participants of the session (n = 1709). | Participation was solicited from associations (n = 20) selected from members of the coordinating committee. These associates participated in recruitment and dissemination of intervention. | Clinicians  Single  Face-to-face workshops to train investigator educators to present the JNC7 framework for blood pressure control, describe the results of the ALLHAT trial, make the results relevant to personal practice, model the use of the guidelines and results, actively encourage use and stimulation questions and discussion. The change methods taught were: persuasion by presenting two-sided arguments and eliciting reservations of participants, role modeling from a respected peer or opinion leader, provision of information and cues to action through materials given to providers at presentations. | The investigator educators who participated (n = 147) reported 1698 presentations to more than 18,500 participants in both pilot and full implementation across 41 states and the District of Columbia from September 2004 to March 2007. Average attendance was 10.9 persons.  The pre- and post-questionnaire indicated positive response to the intervention. |
| Ducharme  2009 [75]  Canada  Alzheimer`s disease  Multiple (qualitative interviews, randomized trial) | Needs assessment  Semi-structure interviews were conducted with caregivers (n = 13) to explore their needs in-depth.  Program objectives  Fostering knowledge and skills required to ensure successful transition to the caregiver role.  Theoretical framework  Model of Role Transition; Illness Beliefs Model; and Social Cognitive Theory.  Designing the Program  The program proposal was developed and then validated through a 90-minute, individual workshop with 11 of the caregivers who participated in the needs assessment. This workshop consisted of a discussion centred on the relevant and clarity of each of the themes covered, as well as on their objectives, the types of activities to be proposed, and the number and frequency of sessions.  Program was tested and qualitatively evaluated with caregivers (n = 2) who had not participated in any of the previous steps of the study to assess the acceptability and feasibility of the intervention and the relevance of its components; the practitioner offered the program meetings; the practitioner completed an evaluation grid in the form of a log to ensure that the meetings took place according to the protocol; the caregivers received a copy of the caregiver workbook; a semi-structured interview grid was then conducted with the caregivers to qualitatively evaluate the program. Changes were made to the program based on the findings.  Implementation planning  Two workbooks were developed, one for the program leader and the other for the caregivers. The second workbook contained documents and exercises to complete between sessions.  Evaluation planning  A randomized controlled trial was planned to assess the effectiveness of the intervention. | 11 of the caregivers that participated in the needs assessment also participated in the validation of the program proposal; the program was mailed to the caregivers, who then met for a 90-minute individual workshop. | Patients  Single  An intervention program for Alzheimer Family Caregivers following diagnostic disclosure: seven individual sessions weekly aimed at fostering knowledge and skills required to ensure successful transition to the caregiver role. | Results of the randomized controlled trial NR. |
| Ramirez-Garcia  2009 [76]  Canada  Antiretroviral treatment  Observational study | Needs assessment  Literature review to identify predictors of optimal treatment taking; partnership with COCQ-Sida (a coalition of community organizations involved in the struggle against AIDS in Quebec); a longitudinal study of people living with HIV/AIDS (n = 376) in the target community was conducted (published elsewhere). The needs assessment culminated with the definition of the ultimate objective of the intervention.  Program objectives  Optimal antiretroviral treatment taking among people living with HIV/AIDS in the community.  Theoretical framework  Bandura's Self Efficacy Theory from Social Cognitive Theory; Elaboration Likelihood Model; and the philosophy of Empowerment.  Designing the Program  Different tools were utilized to facilitate the use of the skills acquired, such as a chart for observing behaviours and a personal diary for recording side-effects. Various documents were used to assure retention of the new information such as leaflets on the range of antiretroviral treatments available and leaflets recommending ways of dealing with adverse side-effects.  The content of the intervention and documentation were validated by HIV health experts: nurses (n = 2), dieticians (n = 2), a physician, and a pharmacist; both the documentation and the intervention were pre-tested with HIV-positive individuals (n = 4) to ensure that the intervention was acceptable, and it met their needs.  Implementation planning  The COCQ-Sida assisted in contacting a family medicine clinic specialized in sexually-transmitted diseases and HIV/AIDS and a service agreement was reached with the clinic to make the intervention available to the people living with HIV/AIDS in exchange for physical space for carry it out. Presentations were conducted on the content of the intervention to facilitate cooperation with the clinic’s professionals.  Evaluation planning  An experimental disease that compared usual care with direct-contract sessions using a self-reported questionnaire and physiological measures. | Collaboration with nurses, social workers, people who are living with HIV/AIDS and other stakeholders throughout the course of intervention development. | Patients  Multifaceted  The intervention consists of four 45-75 minute, direct-contact sessions with an HIV-expert nurse; in the first session the individual is called upon to develop and reinforce skills that would be useful in taking antiretroviral treatment medication as part of the daily routine; the second session focuses on skills to cope with negative emotions and resolve problems; the third session focuses on mastering social skills and the skills for dealing with health professionals in order to enable individuals to mobilize formal and informal networks to assist with antiretroviral treatment medication taking; the fourth session consists of a final booster session to reinforce the skills that have been imparted. | NR |
| Cote  2008 [77]  Canada  Antiretroviral therapy  Quantitative survey | Needs assessment  Literature review of studies on the factors influencing treatment adherence; a large longitudinal study (n = 376) over a 1-year period to confirm the predictors of adherence to antiretroviral therapy and understand the characteristics of current interventions that increase antiretroviral adherence in this population.  Program objectives  To optimize long-term adherence to antiretroviral therapy for people living with HIV/AIDS.  Theoretical framework  Bandura's Self-Efficacy concept from Social Cognitive Theory; and Cognitive Persuasion.  Designing the Program  Program content was validated by various experts (nurse, physician, pharmacist, nutritionist, patient taking the therapy) by asking them to assess aspects of the intervention for clarity, accuracy, appropriateness, and significance of contents. A similar validation grid was developed to allow participants to express their views on the clarity and significant of the content.  Implementation planning  NR  Evaluation planning  NR | NR | Patients  Single  The program consists of two different ways to help medication adherence: 1) direct support consisting of four 45-minute sessions with a nurse every 2-3 weeks; 2) virtual support consisting of at least four interactive 20-30 minutes sessions with a computer over a 2-month period | NR |
| Fransen  2008 [78]  Netherlands  Overweight and obesity  Multiple (qualitative interviews, qualitative focus groups, and program evaluation) | Needs assessment  Literature review of existing interventions and psychosocial theories; stakeholder interviews conducted with health professionals (n = 18) to determine what goes well and what goes wrong in weight management and provide their view on what the intervention would look like; questionnaire study conducted with stakeholders (n = 18) by discussing with them the list of themes from interviews.  Program objectives  Address overweight and obesity among adult primary care patients.  Theoretical framework  Behaviour Change theories.  Designing the Program  A manual for the flow chart was developed, which provides detailed information on how to use the flow charts; a desktop flipchart and patient education materials were provided.  Pre-test: general practitioners (n = 119) were interviewed individually to inquire about the usefulness, workability, appearance, and clarity of the materials and provide suggestions on how the materials could be improved; two focus group interviews with practice nurses (n = 12) were conducted. Additionally, overweight family members (n = 4) were asked to read the patient education materials and provide feedback.  Implementation planning  NR  Evaluation planning  Pilot test will be conducted to assess the usefulness and workability of the intervention. A process and evaluation study will also be conducted to implement the intervention in Dutch primary care practices. | NR | Clinicians  Single  A screening flow chart for general practitioners intended to help determine whether or not a patient is motivated for weight management guidance. | NR |
